# A census of membrane-bound and intracellular signal transduction proteins in bacteria: Bacterial IQ, extroverts and introverts

**DOI:** 10.1186/1471-2180-5-35

**Published:** 2005-06-14

**Authors:** Michael Y Galperin

**Affiliations:** 1 National Center for Biotechnology Information, National Library of Medicine, National Institutes of Health, Bethesda, MD 20894, USA

## Abstract

**Background:**

Analysis of complete microbial genomes showed that intracellular parasites and other microorganisms that inhabit stable ecological niches encode relatively primitive signaling systems, whereas environmental microorganisms typically have sophisticated systems of environmental sensing and signal transduction.

**Results:**

This paper presents results of a comprehensive census of signal transduction proteins – histidine kinases, methyl-accepting chemotaxis receptors, Ser/Thr/Tyr protein kinases, adenylate and diguanylate cyclases and c-di-GMP phosphodiesterases – encoded in 167 bacterial and archaeal genomes, sequenced by the end of 2004. The data have been manually checked to avoid false-negative and false-positive hits that commonly arise during large-scale automated analyses and compared against other available resources. The census data show uneven distribution of most signaling proteins among bacterial and archaeal phyla. The total number of signal transduction proteins grows approximately as a square of genome size. While histidine kinases are found in representatives of all phyla and are distributed according to the power law, other signal transducers are abundant in certain phylogenetic groups but virtually absent in others.

**Conclusion:**

The complexity of signaling systems differs even among closely related organisms. Still, it usually can be correlated with the phylogenetic position of the organism, its lifestyle, and typical environmental challenges it encounters. The number of encoded signal transducers (or their fraction in the total protein set) can be used as a measure of the organism's ability to adapt to diverse conditions, the 'bacterial IQ', while the ratio of transmembrane receptors to intracellular sensors can be used to define whether the organism is an 'extrovert', actively sensing the environmental parameters, or an 'introvert', more concerned about its internal homeostasis. Some of the microorganisms with the highest IQ, including the current leader *Wolinella succinogenes*, are found among the poorly studied beta-, delta- and epsilon-proteobacteria. Among all bacterial phyla, only cyanobacteria appear to be true introverts, probably due to their capacity to conduct oxygenic photosynthesis, using a complex system of intracellular membranes. The census data, available at , can be used to get an insight into metabolic and behavioral propensities of each given organism and improve prediction of the organism's properties based solely on its genome sequence.

## Background

All living organisms adjust their metabolism and behavior in response to the changes in their environment. For unicellular microorganisms, knowing themselves, i.e. constantly monitoring a variety of environmental and intracellular parameters, is a necessary condition of survival. Mechanisms of some adjustments can be as simple as those in the *lac *operon – the presence of a substrate induces expression of the genes that are necessary for assimilation of that substrate (although even *lac *operon has a complex high-level regulation through catabolite repression and inducer exclusion, see [1] and references therein). More complex regulatory mechanisms include transmission of an external signal across the cytoplasmic membrane, followed by intracellular signal transduction to the appropriate genes (operons), metabolic enzymes, or to such organelles as bacterial flagella. Given that all these mechanisms have to be encoded in the organism's genome, the complexity of the signaling systems correlates with the genome size and the range of environmental challenges it normally encounters. Bacterial parasites that inhabit relatively stable host environments typically encode few, if any, signaling proteins (see [2-4]).

**Figure 1:**
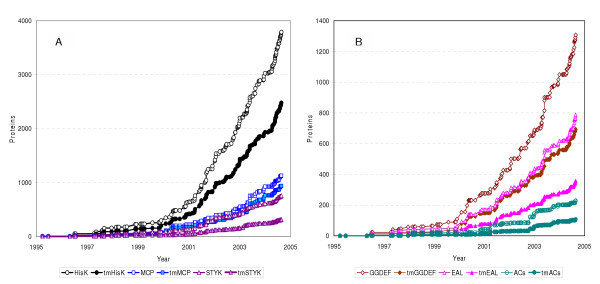
**Growth in the number of signal transduction proteins encoded in complete microbial genomes. **A. Histidine kinases (circles), methyl-accepting chemotaxis proteins (MCPs, squares) and Ser/Thr/Tyr protein kinases (STYKs, triangles). B. Diguanylate cyclases (GGDEF domains, diamonds), c-di-GMP-specific phosphodiesterases (EAL domains, triangles), and adenylate cyclases (circles). Open symbols indicate the total number of proteins with the corresponding domains, closed symbols – the number of membrane-bound proteins of each kind.

Analysis of the first three sequenced microbial genomes revealed very few signaling systems: four histidine kinases (HKs), five response regulators (RRs) and no methyl-accepting chemotaxis proteins (MCPs) in *Haemophilus influenzae*, none of these in *Mycoplasma genitalium *or *Methanococcus *(recently renamed *Methanocaldococcus*) *jannaschii*. Analysis of the fourth sequenced organism, the freshwater cyanobacterium *Synechocystis *sp. PCC 6803, revealed 42 HKs and 38 RRs [5], whereas the fifth, *Mycoplasma pneumoniae*, again had none. The list of signaling proteins encoded in microbial genomes grew by leaps and bounds ever since, generally following the exponential increase in the number of completely sequenced genomes and the total number of proteins that they encode (Figure 1). Given the importance of two-component signal transduction in bacteria [6,7], the numbers of HKs and RRs were routinely reported in many genome descriptions. However, due to the limitations of employed algorithms and arbitrarily high cut-off values in most sequence comparison protocols, certain HK variants were often missed, for example, the HKs of the LytS family (family HPK8 in the classification of Grebe and Stock [8,9]). Some HKs of the recently described HWE family [10] have not been recognized as HKs even when compared against SMART [11,12] and Pfam [13,14] domain databases [15]. Because of that, HKs were systematically undercounted: the number of HKs in *E. coli*, first reported to be 28 [16], was then revised upwards to 29 [2,17] and now stands at 30 [18]; [see Additional file 1]). Likewise, the number of HKs encoded by *Synechocystis *sp. PCC 6803, originally estimated to be 42 [5], has been revised to 46 [2]. As a result, most estimates of the HK numbers published in previous years are unreliable. Besides, listings of signal transduction proteins typically did not take into account Ser/Thr/Tyr-specific protein kinases (STYKs) and protein phosphatases, which, as we now know, were encoded in the *H. influenzae*, *M. genitalium*, and *M. jannaschii *genomes [see Additional file 1], see [19,20]. Further, cross-genome comparisons revealed entirely new classes of signaling molecules with GGDEF and EAL domains, involved in the turnover of the c-di-GMP, a novel secondary messenger [21,22]. Although genetic data and sequence considerations have long pointed to the diguanylate cyclase (c-di-GMP synthetase) activity of the GGDEF domain and the phosphodiesterase (c-di-GMP hydrolase) activity of the EAL domain, direct biochemical proof that this is indeed the case has became available only in the past year [23-25], reviewed in [21,22]. Predicted phosphodiesterase activity of the HD-GYP domain [26] has never been experimentally verified. Finally, although participation of cellular adenylate cyclases (ACs) in signal transduction was never in question, class 3 enzymes (AC3s) were recognized as legitimate environmental sensors only last year, when they were shown to function as light receptors modulating motility in cyanobacteria [27,28]. Adenylate cyclases of class 1 and class 2, represented by experimentally characterized proteins from *E. coli *(AC1, [29]) and *Aeromonas hydrophila *(AC2, [30]), are cytoplasmic enzymes of relatively narrow phylogenetic distribution [see Additional file 1] and are not known to function as environmental sensors.

The diversity of the signal transduction systems made careful accounting for all of them a daunting task, further complicated by the paucity of the data on the cellular targets for STYKs [31] and virtual absence of any data on the mechanisms of c-di-GMP-mediated regulation [21,22]. Hence, most signaling protein surveys focused exclusively on certain classes of membrane receptors (HKs and/or MCPs) and RRs [5,16,17,32-34], or on certain organisms, mostly cyanobacteria and actinobacteria [35-38]. Shi, Kennelly and Potts performed a comprehensive survey of STYKs and protein phosphatases [19,20,39], but have not looked at other signaling proteins. Galperin and colleagues [2,26] performed a census of HKs, GGDEF, and EAL domains but never considered STYKs or ACs. Surveys of the MCP and AC3 distribution in complete microbial genomes by Zhulin [40] and Shenoy and Visweswariah [41], respectively, were limited to these protein domains. The information on signaling systems is poorly represented in public databases. While HKs and RRs are covered in the KEGG database [42,43], other signaling systems are not. The SENTRA [44,45]), SMART [11,12] and COG [46,47] databases have a good coverage of the first sequenced genomes but have not been updated in a while, whereas data in other databases, such as Pfam [13,14] or PEDANT [48,49] are generated mostly by automatic means and therefore prone to the biases described above (and also in the Results section).

While preparing recent reviews on signal transduction in bacteria [3,22], the need for comprehensive and reliable data on the distribution of specific signaling systems among different phylogenetic lineages became all too obvious. Since signal transduction systems grow in number and complexity with the genome size and play increasingly important roles in environmental bacteria [3,4], it has become clear that comparative analysis of such systems could provide a useful insight into bacterial behavior [50]. Here I present a comprehensive census of HKs, MCPs, STYKs and ACs, as well as GGDEF, EAL, and HD-GYP domains encoded in complete genomes of 167 bacterial and archaeal species, sequenced by the end of 2004. I hope that availability of these data on a public web site [51], which will be updated as needed, will stimulate further analysis of microbial signal transduction and will lead to a better understanding of microbial behavior in various ecological niches.

## Results

### Scope of the study

Bacterial signaling mechanisms are extremely diverse, ranging from simplest two-domain transcription regulators, such as AraC or LacI, to multi-component signaling cascades that regulate sporulation, flagellar biosynthesis or biofilm formation. Until recently, the term 'signal transduction' has been typically reserved for the two-component systems consisting of a sensor histidine kinase (HK) and a response regulator (RR). In keeping with this tradition, I did not include in this survey single-component transcriptional regulators, whether of AraC type [52] or much more complex NorR type [53] and considered only dedicated signaling systems that consist of more than two individual components. In addition to HKs, these included Ser/Thr protein kinases, adenylate and diguanylate cyclases and two types of predicted c-di-GMP phosphodiesterases, containing, respectively, EAL or HD-GYP domains. Other enzymatic output domains as well as DNA- or RNA-binding response regulators have not been considered here but could be added to the list in the future. Because of the previously noted parallelism between the domain architectures of intracellular signaling proteins (e.g. PAS-GGDEF-EAL) and respective response regulators (e.g. CheY-PAS-GGDEF-EAL) [3], no attempt has been made to distinguish such proteins; they were counted both in the GGDEF and EAL columns. Naturally, such proteins were counted only once to obtain the total number of signaling proteins encoded in any given genome.

The data set included complete bacterial and archaeal genomes sequenced by the end of 2004. While Archaea and Bacteria are generally considered separate domains of life in the prokaryotic world, there are indications that many signal transduction systems in archaea have been acquired from bacteria through lateral gene transfer [2,32]. Hence, for the purposes of this study, domain Archaea was treated as just another bacterial phylum. Owing to the redundancy of the current genome list, only one representative genome per species was used in the analysis, typically the first one to be publicly released. Exceptions included two strains of *Escherichia coli*, K12 and O157:H7 [54,55], and three serovars of *Salmonella enterica*, Typhi, Typhimurium, and Paratyphi [56-58].

### Data validation

The total numbers of copies of each signaling domain encoded in each given genome were estimated in iterative PSI-BLAST [59] searches, using the strict inclusion threshold expect values of 10^-7^–10^-4^, adjusting as necessary. Potential false-positive hits were checked at every step of PSI-BLAST using the CDD Domain viewer [60] and manually removed (unselected) from the hit list for the next iteration of PSI-BLAST. The most typical sources of the false-positive hits were as follows.

***Histidine kinases ***consist of two separate domains, (i) a well-conserved ATPase domain of the GHKL family [61,62], referred to as HATPase_c domain [Pfam:PF02518 ] in the Pfam database [14], and (ii) a less-conserved phosphoacceptor (dimerization) domain, carrying the phosphorylatable His residue [7,63]. The dimerization domains are quite diverse in their sequence and comprise the His Kinase A (phosphoacceptor) domain clan in Pfam, which unifies four individual domain families: HisKA [Pfam:PF00512 ], HisKA_2 [Pfam:PF07568 ], HisKA_3 [Pfam:PF07730 , and HWE_HK [Pfam:PF07536 ]. Due to the great variability of the HisKA domains, the results of PSI-BLAST search are largely determined by the presence of the HATPase_c domain and often include other members of the GHKL family, such as DNA gyrase B and DNA repair protein MutL, as well as anti-sigma F factors (SpoIIAB-like Ser/Thr kinases). Due to the presence of long α-helices in the phosphoacceptor domains, they sometimes show spurious low-complexity hits.

***Methyl-accepting protein (MCP) domain ***(PF00015) [Pfam:PF00015 ] contains long α-helices, which also attract low-complexity hits. However, the extremely high conservation of the (LI)LALNAAIEAARAGExGRGFAVVAxEVR sequence pattern allows a relatively easy recognition of false-positive hits.

***Ser/Thr/Tyr kinase (STYK) domain ***(PF00069) [Pfam:PF00069 ] belongs to the Protein kinase superfamily clan in Pfam [14]. Other members of this clan, such as kinases of kanamycin, streptomycin, methylthioribose, homoserine, choline, and 3-deoxy-D-manno-octulosonic acid (KDO), are often retrieved in PSI-BLAST searches. In fact, the latter enzyme, KDO kinase (product of the *waaP *gene, PF06293 [Pfam:PF06293 ]) often gives much better BLAST scores than certain divergent Ser/Thr kinases. Most of the discrepancies between the data presented here and those in the KinG database [64,65] could be attributed to those false-positive hits. The most common false-negative hits were the putative protein kinases of ABC1/AarF family (PF03109 [Pfam:PF03109 ] or COG0661 []), which are somehow involved in ubiquinone biosynthesis, most likely by regulating this pathway [66]. It should be noted that although members of the ABC1 (activity of bc1) family are sometimes misannotated as ABC transporters or even ABC transporter substrate binding proteins, this appears to be due to a simple misunderstanding, which I have ignored and counted these proteins as protein kinases.

***GGDEF domains ***(PF00990 [] from diverse bacteria have diguanylate cyclase activity [23,24] and are structurally related to the eukaryotic adenylate cyclase (AC3) domains [67]. While PSI-BLAST searches of GGDEF domains rarely produced any false positive hits, many GGDEF-related domains appeared to be inactivated, some were clearly truncated. The latter ones were excluded from the total count. The most interesting example included a conserved family of proteins (COG3887 []) comprising a fusion of a modified (likely inactivated) GGDEF domain and the DHH-family (PF01368 [Pfam:PF01368 ], [68]) phosphoesterase domain. Members of this family are encoded in genomes of most Firmicutes, including tiny genomes of some *Mycoplasma *spp., but their function remains unknown.

***EAL, AC1, AC2, or AC3 domains ***(corresponding to the Pfam entries PF00563 [Pfam:PF00583 ], PF01295 [Pfam:PF01295 ], PF01928 [Pfam:PF01928 ], and PF00211 [Pfam:PF00211 ], respectively) did not return any false-positive hits in PSI-BLAST searches.

***HD-GYP domain ***is a variant of the widespread HD-type phosphohydrolase (PF01966 [Pfam:PF01966 ], [69]) domain that contains a C-terminal subdomain with extra conserved residues [26]. Classical HD domains without the second subdomain often showed up as false-positive hits; these were filtered based on the total length of the BLAST alignment.

Whenever possible, the domain and protein counts were compared to the published data and all discrepancies were manually verified. Thus, this census has identified 92 HKs in *Bradyrhizobium japonicum*, 62 HKs in *Mesorhizobium loti*, and 48 HKs in *Sinorhizobium meliloti *[see Additional file 1], which was much more than 80, 47 and 40 HKs, respectively, recognized in these bacteria in a recent survey [34]. A comparison of the two sets revealed that most of the proteins missing from the HK list by Hagiwara *et al*.[34] comprise a conserved family (COG3920 []) with an unusual HisKA_2 (PF07568 []) dimerization domain, which, however, still contains a conserved His residue, confirming that these proteins are true HKs. This and other comparisons showed that, in most cases, different authors correctly identified the core sets of signaling proteins and most discrepancies could be attributed to the different ways of treating divergent, inactivated and truncated sequences. The approach adopted here was to take a middle ground, not counting clearly truncated and highly diverged sequences but keeping in the list full-length domains that might have had inactivating point mutations. For example, although Gly?Ala and Glu?Ala changes in the GGEE motif of the GGDEF domain have been shown to abrogate its diguanylate cyclase activity, sequences with such changes were still counted as diguanylate cyclases, while the truncated sequences in *Methanococcus kandleri *protein MK0296 [UniProt:Q8TYK1 ], *Aeropyrum pernix *protein APE1864 [UniProt:Q9YAS9 , or in COG3887 [] proteins (see above) were not. Likewise, *Archaeoglobus fulgidus *encodes a family of proteins that have a typical HK domain architecture but lack the HATPase domain. Such truncated sequences were not included in the total count [see Additional file 1] but still listed (marked with asterisks) in the supporting files. Since the signaling protein count was based on the domain count, monster multidomain proteins, combining various output domains, such as the hybrid HK-STYK [UniProt:O32393 ] described in *Spirulina platensis *[70] or the HK-GGDEF combination, found in *Geobacter sulfurreducens *protein GSU3350 [UniProt:Q747B7 ], have been counted more than once.

### General trends

**Figure 2:**
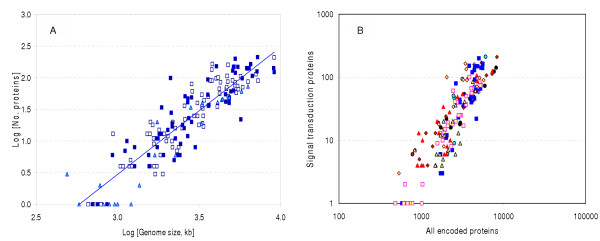
**The total number of encoded signal transducers proteins grows with genome size. **A. Distribution of signal transduction proteins among free-living bacteria and archaea (squares) and obligate pathogens (closed squares). Organisms with other status (symbionts and other commensals) are indicated by triangles. The best-fit line represents data from all species. B. Distribution of signal transduction proteins among organisms of different phylogenetic lineages. The symbols indicate members of the following phyla: Actinobacteria, black circles; Cyanobacteria, open circles (light blue); Alpha-proteobacteria, closed diamonds (dark brown); Beta-, Delta-, and Epsilon-proteobacteria, open diamonds (yellow); Gamma-proteobacteria, closed squares (dark blue); Firmicutes, open squares (magenta); members of other bacterial phyla (Aquificales, Bacteroidetes, Chlamydiae, Deinococcus-Thermus, Planctomycetes, Spirochetes, Thermotoga), closed triangles (red); Archaea, open triangles (yellow).

The census of signal transduction proteins encoded in complete microbial genomes [see Additional file 1] revealed several interesting trends. It has largely confirmed previous observations [2,4,71] that the total number of regulatory proteins encoded by each given organism genome positively correlates with the genome size (Figure 2a) and the total number of encoded proteins (Figure 2b): microbes with complex life styles generally have larger genomes and encode more sophisticated and diverse regulatory systems than parasites with their largely degraded genomes.

While small genome size (and the correspondingly low number of signaling systems) is often associated with pathogenicity, there are numerous pathogens with relatively large genomes (e.g. *Bordetella parapertussis*, *Mycobacterium tuberculosis*), as well as free-living organisms with very small genome sizes (e.g. *Thermoplasma acidophilum*, *Aquifex aeolicus*, see Figure 2a). Many free-living archaea encode very few, if any, proteins involved in signal transduction. For example, among 2977 proteins, encoded by the extreme thermoacidophile *Sulfolobus solfataricus*, only 9 are signaling (8 STYKs and an AC2). A similar picture has been reported in marine cyanobacteria [72] and is seen in the recently sequenced genome of the ruminal bacterium *Mannheimia succiniproducens*, which encodes just 5 HKs, an AC, and a STYK [see Additional file 1]. Apparently, the constant and nutrient-rich ruminal environment does not require much signal transduction. These data indicate that organisms inhabiting stable environments can get away with relatively simple signal transduction systems. In contrast, organisms that survive in diverse ecological niches, including facultative pathogens, such as the spirochetes *Leptospira interrogans *and *Treponema denticola*, typically encode complex sensory systems. Of course, sophisticated bacteria can also be found in simple and stable environments: *Wolinella succinogenes*, another ruminal inhabitant [73], encodes many more signal transduction proteins than other bacteria with similar genome sizes (Table 1, see below).

### Bacterial IQ

The total number of signaling proteins encoded in a given genome (or, rather, the fraction of such proteins among all encoded in the genome) can be used as a measure of the adaptive potential of an organism, some kind of 'bacterial IQ'. The slope of the best-fit line on Figure 2a is 2.03, meaning that the total number of signal transduction proteins grows approximately as a square of the genome size. The organisms whose genomes deviate most from this trend can be considered particularly 'smart' or 'dumb' compared to their relatives. There could be different ways to evaluate the relative abundance of signal transduction proteins at the given genome size; the data in Table 1 were calculated using the following formula:

IQ = 5 × 10^4 ^(n-5)^1/2 ^L^-1^,

where n is the total number of signal transduction proteins, L is the complete genome size in kb (even counting plasmids, it is a more consistent measure than the number of predicted proteins), 5 × 10^4 ^and 5 are arbitrarily chosen empirical coefficients, so that IQ = 100 corresponds to 9 signal transducers in a 1000 kb genome and to 105 transducers in a 5000 kb genome. Accordingly, the IQ value is not defined for organisms with less than 6 signal transduction proteins.

With one exception, all organisms listed in Table 1 are environmental gram-negative bacteria (most gram-positive bacteria and archaea scored much lower) that are highly motile and are known to use a wide variety of electron donors and electron acceptors [73-76]. Such versatile organisms as *Chromobacterium violaceum, Desulfovibrio vulgaris*, *Geobacter sulfurreducens*, *Vibrio vulnificus*, and *Wolinella succinogenes *are also repeatedly found among the leaders in individual categories (Table 2), both in terms of absolute number of signal transduction proteins and of their fraction among all encoded proteins. Remarkably, most of the winners come from the relatively poorly characterized beta-, delta- and epsilon- subdivisions of Proteobacteria. This illustrates the limitations of relying just on *Escherichia coli *and *Bacillus subtilis *as model organisms for studying signaling transduction in environmental organisms. The recent efforts on the post-genomic analysis of the versatile gamma-proteobacterium *Shewanella oneidensis *[77], which encodes a decent set of 46 HKs, 26 MCPs, 7 STYKs, 3 ACs and 52 GGDEF, 28 EAL, and 9 HD-GYP domains [see Additional file 1] might be a step in the right direction. In contrast, *E. coli *appears to have a relatively low IQ. Although its 30 HKs, 19 GGDEF and 17 EAL domains at first seemed like a high number [16,26], *E. coli*, as well as *Salmonella *spp. and *Yersinia *spp., other members of *Enterobacteriaceae*, looks pretty 'dumb' compared to the representatives of *Pseudomonadaceae*, *Vibrionaceae*, or *Xanthomonadaceae*, particularly with respect to chemotaxis: any sequenced member of the three latter families encodes many more MCPs than the meager 5 MCPs in *E. coli*. The deep-sea bacterium *Idiomarina loihiensis*, which belongs to yet another gamma-proteobacterial lineage and whose protein set is just 62% of that of *E. coli *[78], encodes more diguanylate cyclases and 3 times more MCPs than *E. coli*. The delta-proteobacterium *Bdellovibrio bacteriovorus*, a predator that infects an *E. coli *cell and grows in its periplasmic space, also turned out to have a higher IQ: it has a smaller genome than *E. coli *but encodes almost twice as many HKs and four times more MCPs.

### Phylogenetic distribution of signaling systems

**Figure 3:**
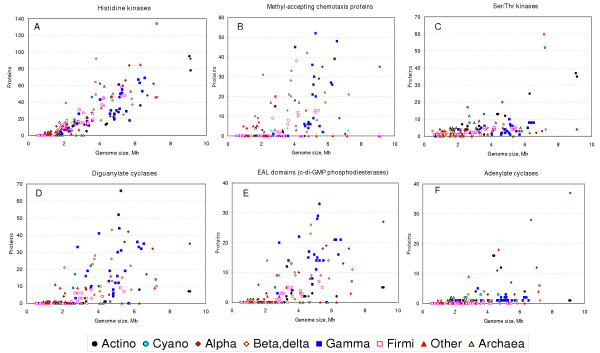
**Phylogenetic distribution of certain types of signal transducers. **A. Histidine kinases. B. Methyl-accepting chemotaxis proteins. C. Ser/Thr/Tyr-protein kinases. D. GGDEF domains (active and inactive diguanylate cyclases). E. EAL domains (active and inactive c-di-GMP phosphodiesterases). F. Adenylate cyclases. The symbols for various phyla are shown at the bottom and are the same as in Fig. 2b.

**Figure 4:**
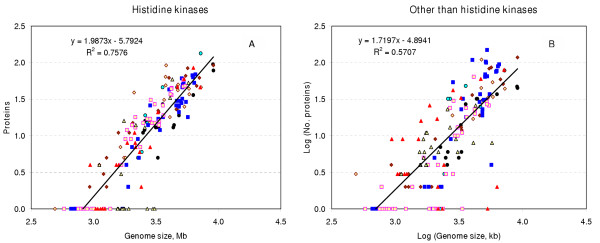
**Distribution of the signal transduction proteins follows the power law. **A. Distribution of histidine kinases. B. Distribution of the total number of all signal transduction proteins except for histidine kinases encoded in a given genome. The symbols for various phyla are as in Fig. 2b.

Histidine kinases are by far the predominant type of sensory proteins (Figure 1), whose distribution in all sequenced organisms generally follows the power law (Figures 3a and 4a). The relative abundance of other types of receptors, however, varies widely among organisms of different phylogenetic lineages (Figure 3b–f). Still, the distribution of their total number also follows power law (Figure 4b). These observations will be analyzed in detail elsewhere. The following is just a brief listing of several unexpected trends:

1. Archaea do not encode AC1- or AC3-type adenylate cyclases, diguanylate cyclases or c-di-GMP-specific phosphodiesterases (with the exception of several highly diverged and probably inactive ORFs), but encode a fair amount of STYKs. In 11 of 20 archaeal genomes, STYKs and class 2 ACs are the only recognizable proteins involved in signal transduction. More than a half of all sequenced archaeal genomes do not encode any MCPs, others encode from 2 to 5 and only the two halophilic species have a large number of MCPs (17 each, Figure 3b).

2. Actinobacteria do not encode MCPs or, for that matter, any other chemotaxis or flagellar proteins (the only one that does, *Symbiobacterium thermophilum*, probably does not belong to the actinobacterial lineage [79]). Instead, actinobacteria encode relatively large numbers of HKs and STYKs (Figure 3a,c). As noted previously, *Mycobacterium tuberculosis *encodes a relatively high number of AC3s [80], as do two other mycobacteria, *M*. *bovis *and *M*. *avium*, but not *M*. *leprae *(16, 16, 12, and 4, respectively). The regulators of these AC3s remain unknown, although some ACs have been implicated in sensing of the bicarbonate level [81]. The dramatically lower number of signaling proteins in *M. leprae*, compared to other mycobacteria, is in line with the general picture of genome decay in this organism [82].

3. Cyanobacteria encode large numbers of HKs and STYKs, but very few MCPs (e.g. 134, 52 and 3, respectively, in *Nostoc *PCC 7120 [see Additional file 1]). These data are consistent with previous observations that cyanobacteria encode just several highly conserved MCPs [37] and regulate their motility using HKs (phytochromes) [83,84] and ACs [27,28].

4. There is great variation between different subdivisions of Proteobacteria with very few common trends. Proteobacteria generally encode few, if any, STYKs, but a large number of MCPs and diguanylate cyclases. The number of ACs is relatively low, except for representatives of the alpha-subdivision. While gamma-proteobacteria typically encode a single AC1 and no more than one AC3, in *Pseudomonas aeruginosa *this sole AC3 is important for virulence [85].

5. Several bacterial phyla that currently have only a handful of sequenced representatives show highly biased patterns of signal transducer distribution. For example, four sequenced members of the Bacteroidetes (formerly the CFB group) encode a relatively large number of HKs (85 in *Bacteroides thetaiotaomicron*), but few or no STYKs and no MCPs, ACs or diguanylate cyclases. It would be interesting to see if this trend holds when more genomes of this lineage become available.

### Variation in IQ between close relatives

The recent genomic data revealed substantial differences in gene content among different strains that, judging by the level of 16S rRNA identity, belong to the same bacterial species [86,87]. It is therefore not surprising to see dramatic differences in signaling protein content among different species of the same genus. Still, different members of the *Bacillus *genus show very similar distributions of signaling proteins [see Additional file 1]. In contrast, three sequenced genomes of *Clostridium *spp. encode dramatically different numbers of MCPs (38 in *C. acetobutylicum*, 20 in *C. tetani *and 0 in *C. perfringens*) and HD-GYP domains (9, 1, and 1, respectively), whereas the content of other signaling proteins is more or less in line with the genome sizes. Accordingly, *C. acetobutylicum *makes it into the winners list in both MCP and HD-GYP categories (Table 2).

Although not seen in the current data set, domains that are missing in one strain were sometimes found in a different strain of the same species. Thus, although this domain census shows the absence of HD-GYP domains in *Yersinia pestis *strain CO92 and in *Bacillus cereus *strain ATCC 14579 [see Additional file 1], this domain is encoded in *Y. pestis *strain KIM and *B. cereus *strain ZK. These differences indicate that signaling proteins can be easily acquired and lost, so all observations on the presence or absence of certain signaling system in a certain organism are only as good as the current genome set.

### Transmembrane and intracellular sensors: Extroverts and introverts

Analysis of complete microbial genomes revealed complex systems of intracellular monitoring that included PAS- and GAF-containing proteins with a variety of output domains [3]. The fraction of membrane-bound proteins among all signal transduction proteins encoded in each given genome was evaluated here using three different methods for predicting transmembrane (TM) segments, followed by manual analysis of the outputs. The census showed that while the great majority of HKs and MCPs were membrane-bound, as much as one-third of all HKs and one-sixth of all MCPs did not contain a single TM segment (Figure 1, Additional file 1). In contrast, only about a half of all adenylate and diguanylate cyclases and c-di-GMP phosphodiesterases were membrane-bound; a majority of STYKs and HD-GYP domains were soluble (Figure 1).

It must be noted that not every membrane-bound signal transduction protein is necessarily a sensor of the environmental parameters. An obvious example among HKs is the turgor sensor KdpD, where TM segments serve solely as anchors [88]. Aer, the energy-sensing MCP, presents a similar case [89]. Conversely, some cytoplasmic sensors might actually sense extracellular signals, e.g. when the sensing domains are present on separate transmembrane polypeptides, as is the case with CheA, the chemotaxis HK. Furthermore, many cytoplasmic sensors respond to signals that are membrane-permeable, such as light, oxygen, H_2_O_2_; NH_3_, and should not be considered purely external or internal. Keeping in mind all these caveats, the predominance of extracellular or intracellular transducers can be used to distinguish organisms that are concerned primarily with sensing environmental parameters ("extroverts") from those more closely monitoring the intracellular milieu ("introverts").

**Figure 5:**
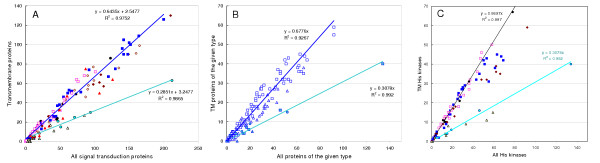
**Phylogenetic distribution of membrane-bound signal transduction proteins. **A. Phylogenetic distribution of the total number of transmembrane signal transducers. The best-fit lines are shown for proteins from gamma-bacteria (dark blue) and cyanobacteria (cyan). The symbols for various phyla are as in Fig. 2b. B. Transmembrane histidine kinases (squares), Ser/Thr kinases (circles) and diguanylate cyclases (triangles) in all proteobacteria (open symbols) and cyanobacteria (closed symbols). The best-fit lines are shown for proteobacterial (dark blue) and cyanobacterial (cyan) histidine kinases. C. Phylogenetic distribution of transmembrane histidine kinases. The best-fit lines are shown for actinobacterial (black) and cyanobacterial (cyan) histidine kinases.

In obligately parasitic bacteria that encode only a handful of signal transduction proteins, most of these proteins are membrane-bound [see Additional file 1]. However, Figure 5a shows that once the total number of signal transduction proteins goes beyond a dozen, the fraction of them that are membrane-bound stabilizes at about 60%, approximately the same in representatives of all phyla, except for Cyanobacteria and Archaea. In the latter two groups, the fraction of membrane-bound signal transduction proteins is close to 30% and also shows very little variance (Figure 5a). Although, as mentioned above, cyanobacteria encode very few MCPs, this fact alone cannot account for the difference between them and all other bacteria. A comparison of other types of signaling proteins encoded in cyanobacteria and proteobacteria (Figure 5b) shows the prevalence of soluble proteins among cyanobacterial HKs, STYKs and GGDEF domains, compared to the prevalence of TM proteins among the same groups of proteins in proteobacteria. The difference between cyanobacterial and archaeal proteins on one hand and proteins from other lineages is most clearly seen in the comparison of HKs (Figure 5c). Remarkably, actinobacteria and firmicutes turn out to be firm extroverts with relatively few intracellular HKs; some of the latter, however, are known to participate in regulation of sporulation [6]. This schism between cyanobacteria and all other bacteria with completely sequenced genomes is likely to be due to the much more complex organization of the cyanobacterial cell, which contains intracellular membranes harboring the photosynthetic reaction centers. Among other autotrophic prokaryotes, prevalence of intracellular proteins is seen in methanogenic archaea and in the green sulfur bacterium *Chlorobium tepidum*, although not in the phototrophic alpha-proteobacterium *Rhodopseudomonas palustris *[see Additional file 1]. Since archaeal heterotrophs also show low amounts of TM signaling proteins, there does not seem to be a direct connection between 'introvertness' and autotrophic metabolism.

## Discussion

This paper has grown out of a survey of signal transduction systems in several alpha- and gamma-proteobacteria prepared for a recent review (Table 1 in ref [3]). It turned out that mere 'counting the senses' could help understand bacterial behavior. For example, as discussed earlier, genomes of two alpha-proteobacteria, *Caulobacter crescentus *and *Mesorhizobium loti*, encode the same number of HKs but the former one encodes 19 MCPs compared to just one in *M. loti *[see Additional file 1]. In contrast, *M. loti *encodes 13 copies of AC3, compared to just two of them in *C. crescentus *([3], [see Additional file 1]). Such observations could provide a useful insight into the physiology of many obscure bacteria whose genomes have been sequenced in the last several years or will be sequenced in the near future. I have therefore updated our previous listing of signal transduction proteins encoded in microbial genomes [2] to cover the genomes sequenced in the past five years.

### Defining the set of signaling proteins

For the purposes of this study, the set of surveyed signal transduction proteins has been limited to just 7 classes of proteins: histidine kinases, methyl-accepting chemotaxis receptors, Ser/Thr protein kinases, adenylate and diguanylate cyclases, c-di-GMP phosphodiesterases with the EAL domain and predicted phosphodiesterases with the HD-GYP domain [see Additional file 1]. Certainly, this list is far from being complete. In a general sense, any cellular protein that participates in cellular adaptation to the changing environment can be considered part of the signaling machinery. Thus, AraC-type transcription regulator, whose DNA-binding properties are modulated by arabinose binding to its N-terminal domain [52], could also be treated as an intracellular signal transducer. According to a recent study by Ulrich, Koonin, and Zhulin, such 'one-component' signalers comprise a majority of signal transduction systems and were the first to arise in evolution [90]. More sophisticated mechanisms of signal transduction include two-component (HK and RR) signal transduction systems and a variety of other signaling systems that have been described only in the past several years (see [2,3,21,22,39] for reviews).

This census considered only dedicated signaling systems that consist of more than two individual components. Therefore, transcriptional regulators, even those of complex domain architecture, were left out (for a comprehensive survey of helix-turn-helix-type (HTH) transcriptional regulators, see [91]). I have also left out response regulators, which are typically considered together with HKs. One of the reasons for that was the frequent confusion between three classes of response regulators: (i) the single-domain chemotaxis response regulator CheY that transmits the signal through protein-protein interactions; (ii) the DNA-binding response regulators of the CheY-HTH domain architecture, and (iii) the response regulators with CheY-AC, CheY-GGDEF or CheY-GGDEF-EAL domain architectures, which produce secondary messengers, cAMP and c-di-GMP. Here, various proteins containing AC, GGDEF, EAL or HD-GYP domains have been lumped together, just as the chemotaxis signal transduction kinase CheA is typically treated as sensor kinase, despite being just a transmitter in the signaling cascade going from MCPs to the flagellar motor. This approach differed from that of Ulrich *et al*. [90], who included diguanylate cyclases and c-di-GMP phosphodiesterases (GGDEF and EAL domains, respectively) into the 'one-component' set.

Another important omission in this survey are Ser/Thr protein phosphatases, which can dephosphorylate STYKs, modulating their activity, and should also be able to dephosphorylate the cellular targets of STYKs. However, several surveys of these enzymes have been published recently [19,39,92], and more are apparently on the way. Due to the difficulties in separating true protein phosphatases from phosphatases of other specificities that often produce false-positive hits I have chosen to exclude them from this survey. Several other systems of the bacterial signal transduction machinery have also been left out. These include (i) Ser/Thr kinases of the bacterial (GHKL) type that regulate the activity of the RNA polymerase sigma subunit; (ii) HPr^Ser ^kinase/phosphorylase and other components of the bacterial PEP-dependent phosphotransferase systems, which regulate chemotaxis, membrane transport (inducer exclusion), and catabolite repression; (iii) the systems that regulate RNA and protein degradation; and many others. A census of each of these systems could be an interesting project in its own right.

The limited scope of this survey, which did not include the sophisticated sporulation machinery of the firmicutes and certain unique (potentially signaling) archaeal domains, could be a reason why representatives of these two groups have generally scored low in the IQ category. Including those proteins into a future version of this census might partly correct that bias, although that would increase the degree of 'introvertness' among archaea even further.

### Caveats of automated domain counting

Even within the limited scope of this survey, there is a lot of space for controversy. There are no clear criteria to decide which proteins should be considered HKs or STYKs and which should be not. Thus, the discrepancies of the results presented here and in the papers by the Mizuno group [5,16,34] can all be attributed to their more conservative approach to defining HKs. The survey by Kim and Forst [17] shows a similar undercount of non-canonical HKs. In contrast, counting STYKs in the KinG database [64] used more permissive criteria than those employed here, which resulted in KDO kinases and other related kinases being counted as STYKs. For other signaling domains, there was much less room for disagreement. The counts of MCPs and ACs, presented here, are very similar to those reported, respectively, by Zhulin [40] and Shenoy and Visweswariah [41]. All our data with supporting information are available on a public web site [51], which should provide an easy way to analyze any discrepancies and, if necessary, correct the final count.

### Do numbers really matter?

It is well known that growth in bacterial genome size is accompanied by accumulation of paralogous protein families, which can be easily seen in lineage-specific expansions of transcriptional regulators, metabolic enzymes, and/or surface proteins [93-95]. It can be argued therefore that the sheer number of signal transduction proteins encoded in a bacterial genome is hardly a good measure of its IQ, as many of these proteins are closely related paralogs. It would seem, however, that lineage-specific expansions that have been fixed in evolution must be of some value to the host organism. Among metabolic enzymes, there are indications of functional diversification even among close paralogs [96]. As for signaling proteins, Valley Stewart and colleagues have shown that NarQ and NarX, two paralogous HKs in *E. coli*, have similar but non-identical functions in modulating cellular response to nitrate and nitrite [97,98]. Likewise, out of 12 GGDEF domain-containing proteins – potential diguanylate cyclases – encoded in *Salmonella *Typhimurium genome, one, AdrA, was found to be primarily responsible for regulating biofilm formation in a complex medium, whereas another, STM1987, was critical for biofilm formation in the nutrient-poor medium [99,100]. These data show that we should be very careful in assigning the same function even to closely related paralogs. Differential regulation of expression and activity of paralogous signal transduction proteins could be yet another sophisticated mechanism allowing the bacterial cell to fine-tune its response to environmental changes. Therefore, until there is clear evidence that functions of paralogous signal transduction proteins are truly identical, the total number of such proteins remains the best measure of the bacterial IQ.

### Intracellular signaling

One of the most significant insights to emerge from comparative genome analysis was the recognition of the vast system of intracellular signaling in bacteria. It became clear that many bacteria encode complex systems of intracellular monitoring whose domain organization is very similar to that used in transmembrane signaling: a sensor domain (typically, PAS and/or GAF), followed by HK, AC, GGDEF or EAL output domains [3]. In certain cases, soluble HKs, MCPs, and ACs have been experimentally characterized and shown to be involved in monitoring levels of intracellular ATP, oxygen, CO, bicarbonate, nitrate, reactive nitrogen species, and other metabolites and modulating the cellular response to the changes in these parameters [101-105]. Some intracellular sensors appeared to be specifically geared towards unusual substrates used by the particular bacterium, such as methanol and formaldehyde in *Paracoccus denitrificans *and *Methylobacterium organophilum *[106,107]. In the recently sequenced genome of *Dehalococcoides ethenogenes*, a major detoxifier of chlorinated organic pollutants, many soluble HKs were found encoded in close proximity to the genes for reductive halogenases, the enzymes that catalyze the dechlorination reactions [108]. It was proposed that these HKs respond to intracellular rather than extracellular stimuli, stimulating the expression of reductive halogenases in response to the presence of their chlorinated substrates [108].

This census shows that intracellular signal transduction proteins comprise a significant fraction of all signal transducers encoded in almost any bacterial genome. However, most of them are still uncharacterized and have yet to be recognized as legitimate members of the bacterial signaling network. The finding that these proteins are abundant in many pathogenic as well as free-living bacteria should help focus the attention of the research community on these novel components of the signal transduction network.

The predominance of intracellular signal transduction proteins in cyanobacteria is in stark contrast with the far smaller proportion of such proteins in other bacterial lineages. There could be several possible reasons for this 'introvertness', all linked to the ability of cyanobacteria to conduct oxygenic photosynthesis. Firstly, cyanobacteria harbor a complex system of intracellular membranes carrying the photosynthetic reaction centers. Intracellular signaling proteins could be needed to control formation and functioning of the photosynthetic system, as well as the transition from phototrophic to heterotrophic metabolism and back. The compartmentalization of the cellular interior probably requires a sophisticated system of monitoring conditions within the individual compartments. Last but not the least, cyanobacteria are unique among (known) prokaryotes in that their cells generate oxygen, which other bacteria try to keep outside the cell. The presence of oxygen affects the redox balance in the cytoplasm and leads to oxidative damage of numerous cellular compounds, including ATP, methionine, cysteine, and many others. It is very likely that numerous intracellular HKs that contain PAS domains are involved in maintaining the constant level of the redox potential in the cyanobacterial cell. Surprisingly, *Rhodopseudomonas palustris*, an alpha-proteobacterium that is also capable of transition between autotrophic and heterotrophic metabolism, does not appear to be an 'introvert' [see Additional file 1]. Hence, it seems that the trend of autotrophic bacteria and archaea being more of 'introverts' and heterotrophic bacteria being more of 'extroverts' might be biased by the current selection of the completely sequenced genomes. It would be interesting to see whether this trend holds when more genomes of bacterial photo- and chemolitotrophs become available.

### Phylum-specific bias and evolution of signal transduction

The knowledge of the phylogenetic distribution of signal transduction systems allows a better understanding of their evolution. Previous analysis of HKs and RRs by Koretke and colleagues led to the conclusion that two-component systems originated in bacteria and radiated into two other domains of life through multiple events of horizontal gene transfer [32]. HKs and STYKs appear to be the principal signal transduction proteins in archaea, suggesting that these two classes of proteins could be already present in the last common ancestor of all living organisms (LUCA, [92,109,110]). The absence of AC3-type adenylate cyclases, diguanylate cyclases and c-di-GMP phosphodiesterases in any of the sequenced archaeal genomes is quite remarkable. In fact, the only full-size archaeal AC3 domain known to date has been found in an uncultivated psychrophilic crenarchaeote that exhibited numerous cases of horizontal gene transfer [111]. Most archaea, however, encode ACs of class 2 (COG1437 []), which are found in only a handful of organisms outside Archaea [30]. These data show that although cAMP is a truly universal second messenger, different domains of life utilize different enzymes for its production and probably employ entirely different mechanisms of cAMP-dependent signaling.

Another remarkable example is the diversity of outputs of the chemotaxis machinery. Although all MCPs counted in this work are very similar, it has been noted [112] that chemotactic signals in diverse bacteria and archaea are being transduced to at least three different motility apparata: the bacterial flagellum, the archaeal flagellum that is unrelated to the bacterial one [113], and to the type IV pili, which are responsible for gliding motility of cyanobacteria and certain other bacteria [84,114].

In general, variability of signal transduction protein content in closely related bacteria, uneven distribution of these proteins among well-established phylogenetic lineages, and the presence in many genomes of tight clusters of closely related paralogs indicate that signaling proteins can be easily acquired and lost. Lineage-specific gene duplication and gene loss and lateral gene transfer probably play a key role in shaping the signaling protein repertoire of each given organism. Why, then, would the total number of signal transduction proteins grow as a square of the genome size (Figure 2a) across a wide variety of microorganisms with diverse lifestyles, phylogenetic affinities, and metabolic capabilities? It is tempting to suggest that there must be an underlying mechanism supporting this correlation. For example, the power-law distribution of HKs (Figure 4a) might stem from the simple fact that the number of binary interactions grows as a square of the number of interacting components [115], so that the number of sensory proteins that manage the linearly growing number of metabolic enzymes has to grow as a square of that number. This explanation is somewhat similar to the one offered by van Nimwegen to explain his observation that the number of transcriptional regulators in bacteria also grows as a square of the genome size [71], although his analysis did not include two-component systems. This was also the rationale behind the decision to measure bacterial IQ as a square root, rather than a linear function, of the total number of encoded signal transduction proteins (see the Results section). However, HKs comprise only but a half of all signal transduction proteins counted in this work [see Additional file 1]. The distribution of other types of signal transducers is even more fascinating: while distribution of each individual class of proteins seems almost random (Figures 3b–f), their ***total ***number still grows approximately as a square of genome size (Figure 4b). One could speculate that this quadratic dependence determines a near-optimal number of signal transducers at a given genome size. This would mean that during their adaptation to different ecological niches, bacteria evolve to rely primarily on certain types of signal transduction, while other types of transducers can be lost (or not fixed in the genome when acquired by lateral gene transfer). For example, during the reductive evolution of chlamydia, HKs and STYKs were retained, while all other transducers and were lost [see Additional file 1]. In contrast, spirochetes held on to their chemotaxis transducers but mostly lost their STYKs. The recent evidence for non-canonical roles of signal transduction proteins, e.g. regulation of gene expression by the chemotaxis system [116] and regulation of chemotaxis by adenylate cyclases [28], suggests that there is certain flexibility in functions of different transducers that could be used by bacterial evolution to generate even greater diversity of signal transduction mechanisms.

### Future developments

The goal of genome analysis is to predict the organism's physiology and behavior based solely on the genomic sequence. There has been great progress in predicting metabolic pathways [110,117,118]; deciphering signaling pathways so far has lagged behind. Accumulation of complete genome sequences has led to the delineation of many new signaling and signal transduction domains and caused a revolution on our understanding of bacterial regulatory networks [2,3,20,119].

I believe that, despite all its limitations, this census would be useful for microbiologists, at least by highlighting still unresolved problems in prokaryotic signal transduction. This work should be complemented by surveys of other components of the signal transduction machinery, including various response regulators, Ser/Thr protein phosphatases, PTS proteins, and many others. Genomes of several environmental microorganisms, including 9-Mb genomes of *Myxococcus xanthus, Rhodococcus *sp., and *Gemmata obscuriglobus*, have been completed and are expected to be publicly released in the near future. Owing to their sheer size, these genomes are likely to bring new signaling domains and illuminate even more regulatory relations. *Myxococcus xanthus*, which reportedly encodes close to 200 HKs and many STYKs, would probably become a leader in both these categories.

The example of *M. xanthus *exposes certain flaws in the IQ calculation method used in this work. This bacterium has extremely complex behavioral patterns [114], but, at 9.1 Mb, it would need to encode more than 550 signal transduction proteins just to make it into the winners' list (Table 1). Certainly, better ways to evaluate bacterial IQ are needed, but that should be subject of a future work. Still, I believe that in the era of 'systems biology' when cellular metabolic pathways are being routinely modeled on a whole-genome level [50,120] and the cell itself is treated more as a machine with a number of interacting parts [121,122], it is important to keep in mind the real complexity of the signal network encoded in each given prokaryotic genome and have an easy measure of this complexity.

I also hope that this census will help us get a better understanding of the microbial diversity and the unique ways that bacteria use to adapt to changing environment. Such understanding is becoming increasingly important as our earlier methods of controlling bacterial growth with one-size-fits-all wide-spectrum antibiotics show progressively diminishing results.

## Conclusion

Careful accounting of diverse proteins participating in prokaryotic signal transduction shows that the complexity of signaling mechanisms correlates well with the organism's genome size and the size of its proteome. The total number of proteins involved in signal transduction, the number of histidine kinases, and the total number of signal transduction proteins other than histidine kinases all grow as square of the genome size. At the same time, the fractions of the latter proteins – MCPs, STYKs, adenylate and diguanylate cyclases and phosphodiesterases – in the total set vary widely depending on the organism's ecology, metabolic properties, and phylogenetic position. The results of this census are freely available to the public and will be updated and corrected as necessary. The availability of this resource, as well as introduction of the concepts of bacterial IQ, introverts and extroverts among the prokaryotes, should help in achieving a better understanding of the microbial behavior and forces that shape microbial genome evolution.

## Methods

### Data sources

Complete genome sequences of 167 bacterial and archaeal species, sequenced by the end of 2004, were downloaded from the NCBI's Genomes database [123] or searched directly through the NCBI web site. Only one representative genome per species was used, usually the first one to be publicly released, according to the NCBI Genomes database listing. Exceptions were made for *Escherichia coli*, represented by two strains, K12 [GenBank:U00096] and O157:H7 [GenBank:BA000007], and *Salmonella enterica*, represented by three serovars, Paratyphi [GenBank:CP000026], Typhi [GenBank:AL513382], and Typhimurium [GenBank:AE006468]. For *Prochlorococcus marinus*, strain CCMP1375 [GenBank:AE017126] genome was used, the middle-sized one of the three. Among other simultaneously released genomes, *Staphylococcus aureus *N315 [GenBank:BA000018], *Streptococcus thermophilus *CNRZ1066 [GenBank:CP000024], and *Thermus thermophilus *HB27 [GenBank:AE017221] genomes were used.

### A census of histidine kinases

The complete list of histidine kinases was compiled separately for each particular phylum of bacteria from the results of BLAST searches against selected genomes using the NCBI's Genomic BLAST tool [124], followed by iterative PSI-BLAST searches [59]. Typically, the searches used as the query sequence the C-terminal fragment (residues 301–579) of the well-characterized histidine kinase PhoR [UniProt:P23545 ] from *Bacillus subtilis*, which contains both HisKA and HATPase domains [125], and a position-specific scoring matrix (PSSM) derived from an alignment of well-characterized histidine kinases (both available as Supplementary Material). Additional searches against the NCBI's Reference Sequence (RefSeq) database [126,127] were performed through the NCBI BLAST web interface  by limiting the search space to the given phylum (e.g. Actinobacteria [orgn]) and excluding reference sequences of incomplete genomes (srcdb_refseq [prop] NOT srcdb_refseq_model [prop]). The PSI-BLAST searches used strict inclusion threshold expect values of 10^-5^–10^-7 ^(adjusting as necessary) and were iterated until no newly retrieved sequences belonged to HKs. The total numbers of copies of each signaling domain encoded in each given genome were estimated using the "Taxonomy Report" option in the BLAST output. Potential false-positive hits were checked at every step of PSI-BLAST using the CDD Domain viewer and manually removed (unselected) from the hit list for the next iteration of PSI-BLAST. In each case where the HATPase domain was easily recognized but HisKA domain was not, a BLAST2sequences [128] search was performed to check whether the HATPase domain was preceded by a conserved region carrying a conserved His residue. The presence of such His-containing regions would indicate that those questionable proteins (e.g., mlr1749 [UniProt:Q98JW4 ] and other members of COG3920 []) comprise legitimate HKs, contrary to the view of Hagiwara *et al*. [34].

Alternatively, PSI-BLAST searches were run against a local copy of the RefSeq database, using the same query sequence and search parameters with additional filtering against sequences translated from unfinished genomes (ZP_xxxxxxxx entries). The resulting hits were compared against the NCBI Taxonomy database to ensure that they all came from a single organism (only one genome of each bacterial species, usually the first one to be sequenced, was used in this analysis). Similar protocol was used to search for histidine kinases in other bacterial phyla.

### Counting other signaling domains

Owing to the relatively high sequence conservation of the MCP, ACyc, GGDEF, and EAL domains, manual checking of the PSI-BLAST outputs revealed very few false-positive hits. In the case of the two latter domains, many low-scoring proteins had numerous amino acid changes, including ones in the likely active sites (see [2,22,67]). No attempt has been made to sort these domains into active and inactive ones. For the HD-GYP domain, which comprises a typical HD superfamily phosphoesterase domain with a number of additional conserved residues, high-scoring BLAST hits to the standard HD domains were filtered based on the shorter length of those hits.

### Identification of transmembrane receptors

Transmembrane (TM) segments in verified sets of signal transduction proteins from various phylogenetic lineages were predicted using PHDhtm [129] and TMHMM [130] programs. The results were sorted into three bins: TM proteins (≥ 2 TM segments), 1 TM proteins, and soluble proteins, and the discrepancies between predictions of the two programs were manually inspected. Comparison of the results revealed many false-negative assignments, so that prediction of a TM segment by either program typically turned out to be justified. Questionable cases were also checked using the HMMTop [131] program, which, however, produced both false-negative and false-positive predictions of TM segments. Therefore, HMMTop assignments were considered only when supported by either PHDhtm or TMHMM results.

## List of Abbreviations

AC, adenylate cyclase;

AC1, adenylate cyclase class 1;

AC2, adenylate cyclase class 2;

AC3, adenylate cyclase class 3;

c-di-GMP, cyclic dimeric (3',5'-guanosine monophosphate);

EAL, conserved protein domain with the Glu-Ala-Leu sequence motif and c-di-GMP-specific phosphodiesterase activity;

GGDEF, conserved protein domain with the Gly-Gly-(Asp/Glu)-Glu-Phe sequence motif and diguanylate cyclase activity;

HD-GYP, conserved protein domain of the HD phosphohydrolase superfamily with additional highly conserved residues, predicted phosphodiesterase;

HK or HisK, histidine kinase;

MCP, methyl-accepting chemotaxis protein;

STYK, Ser/Thr/Tyr-specific protein kinase

TM, transmembrane.

## Authors' contributions

MYG conceived the study, performed all the calculations and wrote the manuscript.

## Supplementary Material

Additional File 1 Results of the census of membrane-bound and intracellular signal transduction proteins in bacteria in HTML format Click here for file

Additional File 2 Table 1 in HTML format Click here for file

Additional File 3 Table 1 in HTML format Click here for file

## Figures and Tables

**Table 1:** Bacteria with the highest adaptability index ("highest IQ")

**Organism**	**Phylum**^a^	**Signal transducers**	**Genome size, kb**	**IQ**
*Wolinella succinogenes*	Epsilon	99	2,110	230
*Geobacter sulfurreducens*	Delta	165	3,814	166
*Idiomarina loihiensis*	Gamma	80	2,839	153
*Desulfovibrio vulgaris*	Delta	135	3,773	151
*Vibrio cholerae*	Gamma	152	4,033	150
*Thermotoga maritima*	Other	34	1,861	145
*Borrelia garinii*	Spiro	13	987	143
*Vibrio vulnificus*	Gamma	200	5,127	136
*Chromobacterium violaceum*	Beta	160	4,751	131
*Thermosynechococcus elongatus*	Cyano	51	2,594	131

^a ^– Beta, Gamma, Delta and Epsilon indicate the corresponding subdivisions of Proteobacteria; Cyano indicates cyanobacteria; Spiro indicates Spirochetes.

**Table 2:** Bacteria and archaea with the highest proportion of encoded signaling proteins of each type

**Organism**	**Phylum**	**No. proteins (%total)**
**Histidine kinases**		
*Nostoc *sp. PCC 7120	Cyano	134 (2.2%)
*Geobacter sulfurreducens*	Delta	92 (2.7%)
*Bacteroides thetaiotaomicron*	Other	85 (1.8%)
*Rhodopseudomonas palustris*	Alpha	66 (1.4%)
*Desulfovibrio vulgaris*	Delta	64 (1.8%)
*Wolinella succinogenes*	Epsilon	39 (1.9%)
*Haloarcula marismortui*	Archaea	59 (1.4%)
**MCPs**		
*Vibrio vulnificus*	Gamma	52 (1.2%)
*Pseudomonas syringae*	Gamma	48 (0.9%)
*Vibrio cholerae*	Gamma	45 (1.2%)
*Chromobacterium violaceum*	Beta	42 (1.0%)
*Clostridium acetobutylicum*	Firmicutes	38 (1.0%)
*Wolinella succinogenes*	Epsilon	31 (1.5%)
*Halobacterium salinarium*	Archaea	17 (0.6%)
**Ser/Thr protein kinases**		
*Rhodopirellula baltica*	Other	60 (0.8%)
*Nostoc *sp. PCC 7120	Cyano	52 (0.9%)
*Streptomyces coelicolor*	Actino	37 (0.4%)
*Streptomyces avermitilis*	Actino	35 (0.4%)
*Gloeobacter violaceus*	Cyano	20 (0.4%)
*.Thermosynechococcus elongatus*	Cyano	17 (0.7%)
*Sulfolobus tokodaii*	Archaea	12 (0.4%)
**Diguanylate cyclases**		
*Vibrio vulnificus*	Gamma	66 (1.5%)
*Shewanella oneidensis*	Gamma	52 (1.2%)
*Vibrio parahaemolyticus*	Gamma	44 (0.9%)
*Chromobacterium violaceum*	Beta	43 (1.0%)
*Vibrio cholerae*	Gamma	41 (1.1%)
*Idiomarina loihiensis*	Gamma	33 (1.3%)
**Adenylate cyclases**		
*Bradyrhizobium japonicum*	Alpha	37 (0.4%)
*Sinorhizobium meliloti*	Alpha	28 (0.5%)
*Leptospira interrogans*	Spiro	18 (0.4%)
*Mycobacterium bovis*	Actino	16 (0.4%)
*Mycobacterium tuberculosis*	Actino	16 (0.4%)
*Treponema denticola*	Spiro	9 (0.3%)
**HD-GYP domains**		
*Desulfovibrio vulgaris*	Delta	14 (0.4%)
*Vibrio vulnificus*	Gamma	13 (0.3%)
*Chromobacterium violaceum*	Beta	11 (0.2%)
*Thermotoga maritima*	Other	10 (0.5%)
*Desulfotalea psychrophila*	Delta	10 (0.3%)
*Geobacter sulfurreducens*	Delta	10 (0.3%)

a – Beta, Gamma, Delta and Epsilon indicate the corresponding subdivisions of Proteobacteria; Cyano indicates cyanobacteria; Spiro indicates Spirochetes.
