# Supplementary material for: A census of membrane-bound and intracellular signal transduction proteins in bacteria: Bacterial IQ, extroverts and introverts
Source: BMC Microbiol. 2005 Jun 14;5:35. doi: 10.1186/1471-2180-5-35 (PMC1183210; doi:10.1186/1471-2180-5-35)
Supplement: Additional File 1 — Results of the census of membrane-bound and intracellular signal transduction proteins in bacteria in HTML format [file 1471-2180-5-35-S1.html]

Census of bacterial signal transduction proteins

### **Table 1. Signal transduction proteins encoded in bacterial and archaeal genomesa**

|  |  |  |  |  |  |  |  |  |  |  |  |  |  |  |  |  |
| --- | --- | --- | --- | --- | --- | --- | --- | --- | --- | --- | --- | --- | --- | --- | --- | --- |
| **No.** | **Organism name** | **Phylum** | **GenBank entry** | **All proteins** | **HisKin** | **MCP** | **STYK** | **GGDEF** | **EAL** | **HD-GYP** | **AC1** | **AC2** | **AC3** | **Total** | **TM** | **%TM** |
| **Actinobacteria** | | | | | | | | | | | | | | | | |
| 1 | Streptomyces coelicolor | Actino | AL645882 | 8154 | **95\***(84) | 0 | **37**(18) | **7\***(2) | **5**(2) | **1**(1) | 0 | 0 | **1**(0) | **142** | **105** | 74% |
| 2 | Streptomyces avermitilis | Actino | BA000030 | 7671 | **78**(67) | 0 | **35**(16) | **7\***(2) | **5**(2) | **1**(1) | 0 | 0 | **1**(0) | **123** | **86** | 70% |
| 3 | Nocardia farcinica | Actino | AP006618 | 5924 | **36**(31) | 0 | **25**(14) | **8**(2) | **2**(1) | 0 | 0 | 0 | **4**(2) | **73** | **49** | 67% |
| 4 | Mycobacterium avium | Actino | AE016958 | 4350 | **19**(16) | 0 | **12**(7) | **4\***(2) | **3**(1) | **1**(0) | 0 | 0 | **12**(7) | **49** | **32** | 65% |
| 5 | Mycobacterium tuberculosis | Actino | AL123456 | 3927 | **14\***(10) | 0 | **13**(8) | **1**(0) | **2**(0) | 0 | 0 | 0 | **16**(6) | **46** | **24** | 52% |
| 6 | Mycobacterium bovis | Actino | BX248333 | 3920 | **13\***(10) | 0 | **13**(7) | **1**(0) | **2**(0) | 0 | 0 | 0 | **16**(6) | **45** | **23** | 51% |
| 7 | Symbiobacterium thermophilum | Actino | AP006840 | 3337 | **27**(21) | **13\***(13) | **1**(1) | **9**(4) | **3**(2) | **4**(0) | 0 | 0 | 0 | **54** | **39** | 72% |
| 8 | Corynebacterium glutamicum | Actino | BA000036 | 2993 | **13**(12) | 0 | **4**(2) | **1**(1) | **1**(1) | 0 | 0 | 0 | **1**(1) | **19** | **16** | 84% |
| 9 | Corynebacterium efficiens | Actino | BA000035 | 2950 | **13**(13) | 0 | **4**(3) | 0 | 0 | 0 | 0 | 0 | **1**(1) | **18** | **17** | 94% |
| 10 | Propionibacterium acnes | Actino | AE017283 | 2297 | **12**(11) | 0 | **4**(3) | 0 | 0 | 0 | 0 | 0 | 0 | **16** | **14** | 88% |
| 11 | Corynebacterium diphtheriae | Actino | BX248353 | 2272 | **11**(11) | 0 | **4**(2) | 0 | 0 | 0 | 0 | 0 | **1**(1) | **16** | **14** | 88% |
| 12 | Leifsonia xyli | Actino | AE016822 | 2030 | **16**(15) | 0 | **6**(6) | 0 | 0 | 0 | 0 | 0 | 0 | **22** | **21** | 95% |
| 13 | Bifidobacterium longum | Actino | AE014295 | 1727 | **9**(8) | 0 | **7**(6) | 0 | 0 | 0 | 0 | 0 | 0 | **16** | **14** | 88% |
| 14 | Mycobacterium leprae | Actino | AL450380 | 1605 | **5**(4) | 0 | **6**(3) | **3**(1) | **2**(0) | 0 | 0 | 0 | **4**(1) | **19** | **9** | 47% |
| 15 | Tropheryma whipplei | Actino | BX072543 | 783 | **2**(1) | 0 | **4**(4) | 0 | 0 | 0 | 0 | 0 | 0 | **6** | **5** | 83% |
| **Cyanobacteria** | | | | | | | | | | | | | | | | |
| 16 | Nostoc sp. PCC 7120 | Cyano | BA000019 | 6055 | **134**(40) | **3**(2) | **52**(15) | **14**(4) | **7**(2) | **2**(0) | 0 | 0 | **6**(2) | **212** | **63** | 30% |
| 17 | Gloeobacter violaceus | Cyano | BA000045 | 4430 | **46**(16) | 0 | **20**(9) | **1**(0) | 0 | **1**(0) | 0 | **2**(0) | **1**(0) | **71** | **25** | 36% |
| 18 | Synechocystis sp. PCC 6803 | Cyano | BA000022 | 3166 | **46**(16) | **4**(3) | **13**(5) | **22**(5) | **13**(2) | **2**(0) | 0 | 0 | **3**(1) | **94** | **30** | 32% |
| 19 | Synechococcus elongatus PCC 6301 | Cyano | AP008231 | 2525 | **15**(4) | **2**(2) | **8**(4) | **17**(8) | **9**(4) | **2**(0) | 0 | 0 | **2**(2) | **47** | **21** | 45% |
| 20 | Synechococcus sp. WH 8102 | Cyano | BX548020 | 2517 | **6**(2) | 0 | **3**(3) | 0 | 0 | 0 | 0 | 0 | 0 | **9** | **5** | 56% |
| 21 | Thermosynechococcus elongatus | Cyano | BA000039 | 2475 | **19**(6) | **3\***(1) | **17**(7) | **9\***(0) | **5**(0) | **1**(0) | 0 | 0 | **2**(1) | **51** | **15** | 29% |
| 22 | Prochlorococcus marinus CCMP1375 | Cyano | AE017126 | 1882 | **5**(2) | 0 | **3**(2) | 0 | 0 | 0 | 0 | 0 | 0 | **8** | **4** | 50% |
| **Alpha-proteobacteria** | | | | | | | | | | | | | | | | |
| 23 | Bradyrhizobium japonicum | Alpha | BA000040 | 8317 | **92**(59) | **35\***(31) | **4\***(2) | **35**(24) | **27**(17) | **3**(0) | 0 | 0 | **37**(12) | **210** | **130** | 62% |
| 24 | Mesorhizobium loti | Alpha | BA000012 | 7275 | **62\***(36) | **1**(1) | **3**(1) | **32**(17) | **18**(10) | **1**(0) | 0 | 0 | **12\***(1) | **113** | **57** | 50% |
| 25 | Sinorhizobium meliloti� | Alpha | AL591688 | 6205 | **48**(32) | **9**(7) | **2**(0) | **17\***(9) | **13\***(6) | 0 | 0 | 0 | **28**(12) | **107** | **61** | 57% |
| 26 | Agrobacterium tumefaciens | Alpha | AE008688 | 5402 | **53**(32) | **20**(14) | **2**(1) | **27\***(17) | **14\***(10) | **1**(0) | 0 | 0 | **3**(2) | **109** | **69** | 63% |
| 27 | Rhodopseudomonas palustris | Alpha | BX571963 | 4813 | **66**(35) | **29**(25) | **5**(3) | **36**(21) | **18**(9) | **2**(0) | 0 | 0 | **7**(4) | **149** | **90** | 60% |
| 28 | Silicibacter pomeroyi� | Alpha | CP000031 | 4252 | **23**(18) | 0 | **2**(1) | **5**(3) | **2**(1) | 0 | 0 | 0 | **11**(3) | **42** | **25** | 60% |
| 29 | Caulobacter crescentus | Alpha | AE005673 | 3737 | **62**(39) | **18\***(12) | **2**(1) | **10\***(4) | **10**(5) | 0 | 0 | 0 | **3**(1) | **98** | **59** | 60% |
| 30 | Brucella suis | Alpha | AE014291 | 3271 | **21**(17) | 0 | **2**(1) | **8**(6) | **5**(4) | 0 | 0 | 0 | 0 | **33** | **26** | 79% |
| 31 | Brucella melitensis | Alpha | AE008917 | 3198 | **22**(13) | 0 | **2**(1) | **9**(6) | **5**(4) | 0 | 0 | 0 | 0 | **35** | **22** | 63% |
| 32 | Zymomonas mobilis | Alpha | AE008692 | 1998 | **15**(10) | **3**(2) | **1**(1) | **4**(3) | **3**(1) | 0 | 0 | 0 | 0 | **24** | **16** | 67% |
| 33 | Bartonella henselae | Alpha | BX897699 | 1488 | **11**(9) | 0 | **1**(1) | **1**(1) | **1**(1) | 0 | 0 | 0 | 0 | **13** | **11** | 85% |
| 34 | Rickettsia conorii� | Alpha | AE006914 | 1374 | **5**(4) | 0 | **1**(0) | **1**(0) | **1**(1) | 0 | 0 | 0 | 0 | **8** | **4** | 50% |
| 35 | Wolbachia endosym-biont of Drosophila | Alpha | AE017196 | 1195 | **2**(1) | 0 | **1**(1) | **1**(0) | 0 | 0 | 0 | 0 | 0 | **4** | **2** | 50% |
| 36 | Bartonella quintana | Alpha | BX897700 | 1142 | **11**(9) | 0 | **1**(1) | **1**(1) | **1**(1) | 0 | 0 | 0 | 0 | **13** | **11** | 85% |
| 37 | Anaplasma marginale | Alpha | CP000030 | 949 | **3**(3) | 0 | **1**(1) | **1**(0) | 0 | 0 | 0 | 0 | 0 | **5** | **4** | 80% |
| 38 | Rickettsia typhi� | Alpha | AE017197 | 838 | **4**(4) | 0 | **1**(0) | **1**(0) | **1**(1) | 0 | 0 | 0 | 0 | **7** | **5** | 71% |
| 39 | Rickettsia prowazekii� | Alpha | AJ235269 | 835 | **4**(4) | 0 | **1**(0) | **1**(0) | **1**(1) | 0 | 0 | 0 | 0 | **7** | **5** | 71% |
| **Beta-proteobacteria** | | | | | | | | | | | | | | | | |
| 40 | Burkholderia pseudomallei | Beta | BX571965 | 5728 | **46**(36) | **21**(21) | **4**(3) | **10**(8) | **11**(4) | **2**(0) | 0 | **1**(0) | 0 | **90** | **68** | 76% |
| 41 | Ralstonia solanacearum | Beta | AL646052 | 5116 | **44**(37) | **22**(22) | **3**(1) | **22\***(16) | **14**(6) | **2**(0) | 0 | **1**(0) | 0 | **98** | **77** | 79% |
| 42 | Bordetella bronchiseptica | Beta | BX470250 | 4994 | **25**(21) | **8**(7) | **4**(3) | **15**(9) | **9**(5) | **2**(1) | 0 | 0 | 0 | **58** | **44** | 76% |
| 43 | Burkholderia mallei | Beta | CP000011 | 4764 | **37**(27) | **17**(17) | **3**(2) | **8**(7) | **8**(4) | **2**(0) | 0 | 0 | 0 | **71** | **53** | 75% |
| 44 | Azoarcus sp. EbN1 | Beta | CR555306 | 4603 | **42**(28) | **2**(2) | **7**(3) | **26**(11) | **23**(7) | **5**(1) | 0 | 0 | **1**(0) | **87** | **46** | 53% |
| 45 | Chromobacterium violaceum | Beta | AE016825 | 4407 | **50**(32) | **42**(33) | **4**(3) | **43**(20) | **26**(7) | **11**(0) | 0 | 0 | 0 | **160** | **91** | 57% |
| 46 | Bordetella parapertussis | Beta | BX470249 | 4185 | **22**(18) | **6**(6) | **4**(3) | **13**(8) | **9**(5) | **2**(1) | 0 | 0 | 0 | **51** | **38** | 75% |
| 47 | Bordetella pertussis | Beta | BX470248 | 3436 | **18**(16) | **5**(5) | **3**(2) | **7**(3) | **3**(1) | **1**(0) | 0 | 0 | 0 | **35** | **26** | 74% |
| 48 | Nitrosomonas europaea | Beta | AL954747 | 2461 | **15**(11) | **3**(3) | **2**(1) | **6**(3) | **9**(3) | 0 | 0 | 0 | **3**(1) | **34** | **20** | 59% |
| 49 | Neisseria meningitidis | Beta | AE002098 | 2079 | **5**(5) | 0 | **1**(1) | 0 | 0 | 0 | 0 | 0 | 0 | **6** | **6** | 100% |
| **Gamma-proteobacteria** | | | | | | | | | | | | | | | | |
| 50 | Pseudomonas syringae | Gamma | AE016853 | 5608 | **69\***(42) | **48\***(41) | **8**(2) | **35**(18) | **21**(14) | **1**(0) | **1**(0) | 0 | 0 | **164** | **109** | 67% |
| 51 | Pseudomonas aeruginosa | Gamma | AE004091 | 5567 | **63**(45) | **26**(23) | **8**(2) | **33**(19) | **21**(14) | **3**(0) | **1**(0) | 0 | **1**(1) | **140** | **96** | 69% |
| 52 | Photobacterium profundum | Gamma | CR354531 | 5480 | **53**(43) | **39**(37) | **8**(3) | **32\***(18) | **16**(10) | **2**(0) | **1**(0) | **1**(0) | 0 | **142** | **105** | 74% |
| 53 | Pseudomonas putida | Gamma | AE015451 | 5350 | **67**(44) | **27**(22) | **5**(1) | **36**(21) | **21**(11) | **2**(1) | **1**(0) | 0 | 0 | **142** | **90** | 63% |
| 54 | Escherichia coli O157:H7 | Gamma | BA000007 | 5253 | **29**(27) | **5**(5) | **3**(2) | **19**(12) | **14**(10) | 0 | **1**(0) | 0 | 0 | **66** | **51** | 77% |
| 55 | Vibrio vulnificus CMCP6 | Gamma | AE016795 | 5024 | **51**(40) | **52**(43) | **4**(1) | **66**(38) | **33**(14) | **13**(4) | **1**(0) | 0 | 0 | **200** | **126** | 63% |
| 56 | Vibrio parahaemolyticus | Gamma | BA000031 | 4832 | **50**(42) | **30**(26) | **6**(2) | **44\***(27) | **29**(14) | **5**(1) | **1**(0) | **1**(0) | 0 | **150** | **102** | 68% |
| 57 | Salmonella enterica serovar Typhi� | Gamma | AL513382 | 4758 | **30**(28) | **6**(4) | **3**(1) | **12**(11) | **16**(11) | 0 | **1**(0) | 0 | 0 | **61** | **50** | 82% |
| 58 | Photorhabdus luminescens | Gamma | BX470251 | 4683 | **18**(16) | **2**(2) | **1**(1) | 0 | 0 | 0 | **1**(0) | 0 | 0 | **22** | **19** | 86% |
| 59 | Salmonella typhimurium LT2 | Gamma | AE006468 | 4527 | **32**(29) | **9**(7) | **1**(1) | **12**(11) | **15**(11) | 0 | **1**(0) | 0 | 0 | **63** | **53** | 84% |
| 60 | Erwinia carotovora | Gamma | BX950851 | 4472 | **28**(23) | **36**(36) | **2**(1) | **16\***(11) | **11**(5) | 0 | **1**(0) | **1**(0) | 0 | **92** | **74** | 80% |
| 61 | Shewanella oneidensis | Gamma | AE014299 | 4467 | **46**(35) | **26**(24) | **7**(1) | **52\***(26) | **27\***(14) | **9**(1) | **1**(0) | **1**(0) | **1**(1) | **148** | **90** | 61% |
| 62 | Shigella flexneri | Gamma | AE005674 | 4441 | **25**(22) | **4**(4) | **2**(2) | **8**(6) | **8**(6) | 0 | **1**(0) | 0 | 0 | **46** | **37** | 80% |
| 63 | Xanthomonas axonopodis | Gamma | AE008923 | 4427 | **61**(38) | **21**(18) | **10**(3) | **30**(14) | **14**(5) | **3**(0) | 0 | 0 | **1**(1) | **130** | **75** | 58% |
| 64 | Escherichia coli K12 | Gamma | U00096 | 4242 | **30**(27) | **5**(5) | **2**(2) | **19**(12) | **17**(11) | 0 | **1**(0) | 0 | 0 | **67** | **52** | 78% |
| 65 | Xanthomonas campestris | Gamma | AE008922 | 4181 | **55**(31) | **20**(16) | **6**(3) | **31**(15) | **14**(5) | **3**(0) | 0 | 0 | **1**(1) | **120** | **67** | 56% |
| 66 | Salmonella enterica serovar Paratyphi | Gamma | CP000026 | 4093 | **26**(24) | **6**(4) | **1**(1) | **13**(12) | **15**(11) | 0 | **1**(0) | 0 | 0 | **55** | **46** | 84% |
| 67 | Yersinia pestis | Gamma | AL590842 | 4067 | **21**(19) | **6**(4) | **4**(1) | **5**(5) | **6**(4) | 0 | **1**(0) | 0 | 0 | **40** | **31** | 78% |
| 68 | Yersinia pseudotuberculosis | Gamma | BX936398 | 4038 | **26**(25) | **7**(6) | **4**(1) | **6**(5) | **6**(4) | **1**(0) | **1**(0) | **1**(0) | 0 | **49** | **38** | 78% |
| 69 | Vibrio cholerae� | Gamma | AE003852 | 3835 | **43**(32) | **45**(37) | **1**(1) | **41**(27) | **22**(8) | **9**(2) | **1**(0) | 0 | 0 | **152** | **100** | 66% |
| 70 | Acinetobacter sp. ADP1 | Gamma | CR543861 | 3325 | **18**(14) | 0 | **6**(1) | **10**(6) | **4**(1) | 0 | 0 | 0 | **1**(1) | **36** | **22** | 61% |
| 71 | Methylococcus capsulatus | Gamma | AE017282 | 2959 | **17**(14) | **2**(1) | **3**(2) | **16**(8) | **12**(5) | **1**(0) | 0 | 0 | 0 | **40** | **26** | 65% |
| 72 | Legionella pneumophila | Gamma | AE017354 | 2942 | **12**(7) | 0 | **5**(3) | **19**(11) | **14**(6) | 0 | 0 | 0 | **5**(4) | **44** | **26** | 59% |
| 73 | Xylella fastidiosa 9a5c | Gamma | AE003849 | 2832 | **14**(9) | **1**(1) | **2**(1) | **3**(1) | **3**(1) | **1**(0) | 0 | 0 | 0 | **23** | **13** | 57% |
| 74 | Idiomarina loihiensis | Gamma | AE017340 | 2640 | **26**(18) | **15**(13) | **2**(2) | **33**(17) | **20**(11) | **1**(0) | 0 | 0 | 0 | **80** | **51** | 64% |
| 75 | Mannheimia succiniciproducens | Gamma | AE016827 | 2384 | **5**(5) | 0 | **1**(0) | 0 | 0 | 0 | **1**(0) | 0 | 0 | **7** | **5** | 71% |
| 76 | Coxiella burnetii | Gamma | AE016828 | 2046 | **7**(2) | 0 | **4**(1) | **0\*** | 0 | 0 | 0 | 0 | **1**(0) | **12** | **3** | 25% |
| 77 | Pasteurella multocida� | Gamma | AE004439 | 2015 | **9**(9) | 0 | **1**(1) | 0 | 0 | 0 | **1**(0) | 0 | 0 | **11** | **10** | 91% |
| 78 | Francisella tularensis | Gamma | AJ749949 | 1804 | **2**(2) | 0 | **1**(1) | 0 | 0 | 0 | 0 | 0 | 0 | **3** | **3** | 100% |
| 79 | Haemophilus ducreyi | Gamma | AE017143 | 1717 | **1**(1) | 0 | **1**(1) | 0 | 0 | 0 | **1**(0) | 0 | 0 | **3** | **2** | 67% |
| 80 | Haemophilus influenzae | Gamma | L42023 | 1711 | **4**(3) | 0 | **1**(0) | 0 | 0 | 0 | **1**(0) | 0 | 0 | **6** | **3** | 50% |
| 81 | Wigglesworthia glossinidia | Gamma | BA000021 | 617 | 0 | 0 | **1**(1) | 0 | 0 | 0 | 0 | 0 | 0 | **1** | **1** | 100% |
| 82 | Candidatus Blochmannia floridanus | Gamma | BX248583 | 583 | 0 | 0 | **1**(1) | 0 | 0 | 0 | 0 | 0 | 0 | **1** | **1** | 100% |
| 83 | Buchnera aphidicola str. APS | Gamma | BA000003 | 574 | 0 | 0 | 0 | 0 | 0 | 0 | 0 | 0 | 0 | 0 | 0 | nd |
| **Delta-proteobacteria** | | | | | | | | | | | | | | | | |
| 84 | Bdellovibrio bacteriovorus | Delta | BX842601 | 3587 | **49**(30) | **20**(18) | **5**(1) | **5**(1) | **1**(0) | **6**(0) | 0 | 0 | **4**(4) | **90** | **54** | 60% |
| 85 | Desulfovibrio vulgaris | Delta | AE017285 | 3531 | **64**(36) | **28**(28) | **1**(0) | **26**(11) | **10**(3) | **14**(2) | 0 | **1**(0) | 0 | **135** | **76** | 56% |
| 86 | Geobacter sulfurreducens | Delta | AE017180 | 3446 | **92**(55) | **33**(30) | **1**(1) | **28**(13) | **9**(4) | **10**(0) | 0 | 0 | 0 | **165** | **99** | 60% |
| 87 | Desulfotalea psychrophila | Delta | CR522870 | 3236 | **31**(24) | **21**(20) | **4**(2) | **23**(12) | **4**(2) | **10**(5) | 0 | 0 | **1**(0) | **91** | **58** | 64% |
| **Epsilon-proteobacteria** | | | | | | | | | | | | | | | | |
| 88 | Wolinella succinogenes | Epsilon | BX571656 | 2044 | **39**(22) | **31**(27) | **2**(1) | **21**(10) | **13\***(5) | **2**(0) | 0 | 0 | **1**(1) | **99** | **63** | 64% |
| 89 | Helicobacter hepaticus | Epsilon | AE017125 | 1875 | **5**(3) | **9**(8) | 0 | **1**(0) | 0 | 0 | 0 | 0 | 0 | **15** | **11** | 73% |
| 90 | Campylobacter jejuni | Epsilon | AL111168 | 1634 | **7**(4) | **10**(6) | 0 | **1**(0) | 0 | 0 | 0 | 0 | 0 | **18** | **10** | 56% |
| 91 | Helicobacter pylori | Epsilon | AE000511 | 1576 | **4**(1) | **4**(3) | **1**(0) | 0 | 0 | 0 | 0 | 0 | 0 | **9** | **4** | 44% |
| **Firmicutes** | | | | | | | | | | | | | | | | |
| 92 | Bacillus anthracis str. Ames | Firmi | AE016879 | 5311 | **47**(43) | **13**(11) | **5**(2) | **9\***(6) | **7**(4) | **1**(1) | 0 | 0 | 0 | **76** | **62** | 82% |
| 93 | Bacillus cereus ATCC 14579 | Firmi | AE016877 | 5255 | **48**(46) | **13**(11) | **5**(3) | **7\***(4) | **5**(2) | 0 | 0 | 0 | 0 | **74** | **64** | 86% |
| 94 | Bacillus thuringiensis | Firmi | AE017355 | 5117 | **58**(50) | **12**(10) | **4**(2) | **9\***(6) | **7**(4) | **1**(1) | 0 | 0 | 0 | **85** | **68** | 80% |
| 95 | Bacillus licheniformis | Firmi | AE017333 | 4196 | **36**(27) | **11\***(9) | **3**(1) | **4\***(3) | **2**(0) | 0 | 0 | 0 | 0 | **56** | **40** | 71% |
| 96 | Bacillus subtilis | Firmi | AL009126 | 4105 | **36**(30) | **10**(8) | **4**(3) | **4\***(3) | **3**(1) | 0 | 0 | 0 | 0 | **56** | **44** | 79% |
| 97 | Bacillus halodurans | Firmi | BA000004 | 4066 | **44**(36) | **12**(10) | **4**(3) | **4\***(1) | **2**(0) | **2**(0) | 0 | 0 | 0 | **67** | **50** | 75% |
| 98 | Clostridium acetobutylicum | Firmi | AE001437 | 3848 | **37**(32) | **38\***(30) | **5**(3) | **10\***(7) | **4**(1) | **9**(1) | 0 | **1**(0) | 0 | **101** | **72** | 71% |
| 99 | Geobacillus kaustophilus | Firmi | BA000043 | 3540 | **26\***(18) | **6**(5) | **5**(4) | **8\***(4) | **5**(2) | **3**(1) | 0 | 0 | 0 | **50** | **32** | 64% |
| 100 | Oceanobacillus iheyensis | Firmi | BA000028 | 3500 | **19**(16) | **8**(6) | **4**(3) | **3\***(1) | **2**(0) | **2**(0) | 0 | 0 | 0 | **37** | **26** | 70% |
| 101 | Enterococcus faecalis | Firmi | AE016830 | 3267 | **16**(15) | 0 | **1**(1) | **0\*** | 0 | 0 | 0 | 0 | 0 | **17** | **16** | 94% |
| 102 | Lactobacillus plantarum | Firmi | AL935263 | 3059 | **14**(13) | 0 | **3**(3) | **3\***(3) | **5**(1) | 0 | 0 | 0 | 0 | **25** | **20** | 80% |
| 103 | Listeria innocua | Firmi | AL592022 | 2968 | **15**(13) | **2**(1) | **2**(2) | **3\***(3) | **3**(0) | 0 | 0 | 0 | 0 | **25** | **19** | 76% |
| 104 | Listeria monocytogenes | Firmi | AL591824 | 2846 | **15**(13) | **2**(1) | **2**(2) | **3\***(3) | **3**(0) | 0 | 0 | 0 | 0 | **25** | **19** | 76% |
| 105 | Clostridium perfringens | Firmi | BA000016 | 2723 | **27**(24) | 0 | **3**(2) | **6\***(4) | **3**(2) | **1**(1) | 0 | **1**(0) | 0 | **40** | **31** | 76% |
| 106 | Staphylococcus aureus N315 | Firmi | BA000018 | 2624 | **17**(15) | 0 | **2**(1) | **1\***(1) | 0 | 0 | 0 | 0 | 0 | **20** | **17** | 85% |
| 107 | Thermoanaerobacter tengcongensis | Firmi | AE008691 | 2588 | **17**(12) | **9**(7) | **3**(3) | **6\***(1) | **1**(0) | **6**(0) | 0 | 0 | 0 | **41** | **23** | 56% |
| 108 | Staphylococcus epidermidis | Firmi | AE015929 | 2419 | **15**(13) | 0 | **2**(1) | **1\***(1) | 0 | 0 | 0 | 0 | 0 | **18** | **15** | 83% |
| 109 | Clostridium tetani | Firmi | AE015927 | 2373 | **31**(24) | **20**(16) | **1**(1) | **6\***(4) | **1**(0) | **1**(0) | 0 | **1**(0) | 0 | **61** | **45** | 74% |
| 110 | Lactococcus lactis | Firmi | AE005176 | 2321 | **7**(7) | 0 | **1**(1) | **0\*** | 0 | 0 | 0 | 0 | 0 | **8** | **8** | 100% |
| 111 | Streptococcus agalactiae | Firmi | AE009948 | 2124 | **18**(18) | 0 | **1**(1) | **0\*** | 0 | 0 | 0 | 0 | 0 | **19** | **19** | 100% |
| 112 | Streptococcus pneumoniae TIGR4 | Firmi | AE005672 | 2094 | **13**(13) | 0 | **4**(1) | **0\*** | 0 | 0 | 0 | 0 | 0 | **17** | **14** | 82% |
| 113 | Streptococcus mutans | Firmi | AE014133 | 1960 | **14**(13) | 0 | **1**(0) | **0\*** | 0 | 0 | 0 | 0 | 0 | **15** | **13** | 87% |
| 114 | Streptococcus thermophilus | Firmi | CP000024 | 1915 | **9\***(8) | 0 | **4**(2) | **0\*** | 0 | 0 | 0 | 0 | 0 | **13** | **10** | 77% |
| 115 | Lactobacillus johnsonii | Firmi | AE017198 | 1821 | **9**(9) | 0 | **1**(1) | **0\*** | 0 | 0 | 0 | 0 | 0 | **10** | **10** | 100% |
| 116 | Streptococcus pyogenes M1 GAS | Firmi | AE004092 | 1697 | **12**(12) | 0 | **1**(1) | **0\*** | 0 | 0 | 0 | 0 | 0 | **13** | **13** | 100% |
| 117 | Mycoplasma penetrans | Firmi | BA000026 | 1037 | 0 | 0 | **2**(1) | 0 | 0 | 0 | 0 | 0 | 0 | **2** | **1** | 50% |
| 118 | Mycoplasma mycoides | Firmi | BX293980 | 1016 | 0 | 0 | **1**(1) | 0 | 0 | 0 | 0 | 0 | 0 | **1** | **1** | 100% |
| 119 | Mycoplasma pulmonis | Firmi | AL445566 | 782 | 0 | 0 | **1**(1) | **0\*** | 0 | 0 | 0 | 0 | 0 | **1** | **1** | 100% |
| 120 | Onion yellows phytoplasma | Firmi | AP006628 | 754 | 0 | 0 | 0 | **0\*** | 0 | 0 | 0 | 0 | 0 | 0 | 0 | nd |
| 121 | Mycoplasma gallisepticum | Firmi | AE015450 | 726 | 0 | 0 | **1**(1) | 0 | 0 | 0 | 0 | 0 | 0 | **1** | **1** | 100% |
| 122 | Mycoplasma hyopneumoniae | Firmi | AE017332 | 691 | 0 | 0 | 0 | **0\*** | 0 | 0 | 0 | 0 | 0 | 0 | 0 | nd |
| 123 | Mycoplasma pneumoniae | Firmi | U00089 | 689 | 0 | 0 | **1**(1) | 0 | 0 | 0 | 0 | 0 | 0 | **1** | **1** | 100% |
| 124 | Mesoplasma florum | Firmi | AE017263 | 682 | 0 | 0 | **1**(1) | 0 | 0 | 0 | 0 | 0 | 0 | **1** | **1** | 100% |
| 125 | Mycoplasma mobile | Firmi | AE017308 | 633 | 0 | 0 | **2**(1) | **0\*** | 0 | 0 | 0 | 0 | 0 | **2** | **1** | 50% |
| 126 | Ureaplasma parvum | Firmi | AF222894 | 614 | 0 | 0 | **1**(1) | 0 | 0 | 0 | 0 | 0 | 0 | **1** | **1** | 100% |
| 127 | Mycoplasma genitalium | Firmi | L43967 | 484 | 0 | 0 | **1**(1) | 0 | 0 | 0 | 0 | 0 | 0 | **1** | **1** | 100% |
| **Other bacterial phyla** | | | | | | | | | | | | | | | | |
| 128 | Rhodopirellula baltica� | Other | BX119912 | 7325 | **46\***(22) | 0 | **60**(38) | **9\***(2) | **3**(0) | **6**(0) | 0 | **1**(0) | **3**(2) | **126** | **64** | 51% |
| 129 | Bacteroides thetaiotaomicron | Other | AE015928 | 4778 | **85**(73) | 0 | **2**(2) | 0 | 0 | 0 | 0 | 0 | 0 | **87** | **75** | 86% |
| 130 | Bacteroides fragilis | Other | AP006841 | 4625 | **60**(47) | 0 | 0 | 0 | 0 | 0 | 0 | 0 | 0 | **60** | **47** | 78% |
| 131 | Deinococcus radiodurans | Other | AE000513 | 3184 | **21**(12) | **3**(3) | **9\***(2) | **16**(7) | **5**(0) | **4**(0) | 0 | 0 | 0 | **54** | **24** | 44% |
| 132 | Chlorobium tepidum | Other | AE006470 | 2252 | **8**(2) | 0 | 0 | **1**(1) | 0 | 0 | 0 | 0 | **1**(0) | **10** | **3** | 30% |
| 133 | Thermus thermophilus� | Other | AE017221 | 2210 | **11**(8) | 0 | **4**(1) | **6\***(5) | **2**(0) | **5**(0) | 0 | 0 | **1**(0) | **28** | **14** | 57% |
| 134 | Fusobacterium nucleatum� | Other | AE009951 | 2066 | **7**(6) | 0 | 0 | 0 | 0 | 0 | 0 | 0 | **1**(0) | **8** | **6** | 75% |
| 135 | Parachlamydia sp. UWE25 | Other | BX908798 | 2031 | **2**(1) | 0 | **4**(0) | 0 | 0 | 0 | 0 | 0 | 0 | **6** | **1** | 17% |
| 136 | Porphyromonas gingivalis | Other | AE015924 | 1909 | **6**(6) | 0 | 0 | 0 | 0 | 0 | 0 | 0 | 0 | **6** | **6** | 100% |
| 137 | Thermotoga maritima | Other | AE000512 | 1858 | **8**(4) | **7**(6) | 0 | **9\***(4) | 0 | **10**(2) | 0 | 0 | 0 | **34** | **16** | 47% |
| 138 | Aquifex aeolicus | Other | AE000657 | 1560 | **4**(2) | 0 | **2**(0) | **11\***(4) | **6\***(1) | **1**(0) | 0 | 0 | 0 | **20** | **6** | 30% |
| 139 | Chlamydophila pneumoniae CWL029 | Other | AE001363 | 1054 | **1**(0) | 0 | **3**(0) | 0 | 0 | 0 | 0 | 0 | 0 | **4** | **0** | 0% |
| 140 | Chlamydophila caviae | Other | AE015925 | 1005 | **1**(0) | 0 | **3**(0) | 0 | 0 | 0 | 0 | 0 | 0 | **4** | **0** | 0% |
| 141 | Chlamydia muridarum | Other | AE002160 | 904 | **1**(0) | 0 | **3**(0) | 0 | 0 | 0 | 0 | 0 | 0 | **4** | **0** | 0% |
| 142 | Chlamydia trachomatis | Other | AE001273 | 895 | **1**(0) | 0 | **3**(0) | 0 | 0 | 0 | 0 | 0 | 0 | **4** | **0** | 0% |
| **Spirochetes** | | | | | | | | | | | | | | | | |
| 143 | Leptospira interrogans | Spiro | AE010300 | 4727 | **47**(20) | **13**(13) | **5**(2) | **16**(3) | **8**(0) | **2**(0) | 0 | 0 | **18**(12) | **107** | **50** | 47% |
| 144 | Treponema denticola� | Spiro | AE017226 | 2767 | **7**(3) | **20**(19) | 0 | **9**(4) | **2**(2) | **4**(0) | 0 | 0 | **9**(7) | **49** | **33** | 67% |
| 145 | Borrelia burgdorferi | Spiro | AE001115 | 1639 | **4**(1) | **5**(4) | 0 | **1**(0) | **1**(0) | **1**(0) | 0 | **1**(0) | 0 | **13** | **5** | 39% |
| 146 | Treponema pallidum� | Spiro | AE000520 | 1036 | **1**(0) | **4**(4) | 0 | **1\***(0) | 0 | **3**(0) | 0 | 0 | **1**(1) | **10** | **5** | 50% |
| 147 | Borrelia garinii | Spiro | CP000013 | 932 | **4**(1) | **5**(4) | 0 | **1**(0) | **1**(0) | **1**(0) | 0 | **1**(0) | 0 | **13** | **5** | 39% |
| **Archaea** | | | | | | | | | | | | | | | | |
| 148 | Methanosarcina acetivorans | Archaea | AE010299 | 4540 | **53**(11) | **2**(1) | 5(2) | 0 | 0 | 0 | 0 | **1**(0) | 0 | **61** | **14** | 23% |
| 149 | Haloarcula marismortui | Archaea | AY596297 | 4240 | **59**(15) | **17**(9) | **8**(3) | 0 | 0 | 0 | 0 | **1**(0) | 0 | **85** | **27** | 32% |
| 150 | Methanosarcina mazei | Archaea | AE008384 | 3371 | **33**(9) | **3**(1) | **4**(1) | 0 | 0 | 0 | 0 | **1**(0) | 0 | **41** | **11** | 28% |
| 151 | Sulfolobus solfataricus | Archaea | AE006641 | 2977 | 0 | 0 | **8**(3) | 0 | 0 | 0 | 0 | **1**(0) | 0 | **9** | **3** | 38% |
| 152 | Sulfolobus tokodaii | Archaea | BA000023 | 2825 | 0 | 0 | **12**(6) | 0 | 0 | 0 | 0 | **1**(0) | 0 | **13** | **6** | 50% |
| 153 | Halobacterium salinarum | Archaea | AE004437 | 2622 | **13**(4) | **17**(12) | **5**(1) | 0 | 0 | 0 | 0 | **1**(0) | 0 | **36** | **17** | 49% |
| 154 | Pyrobaculum aerophilum | Archaea | AE009441 | 2605 | 0 | 0 | **5**(1) | 0 | 0 | 0 | 0 | **1**(0) | 0 | **6** | **1** | 20% |
| 155 | Archaeoglobus fulgidus | Archaea | AE000782 | 2420 | **14\***(3) | **2**(0) | **3**(0) | 0 | 0 | 0 | 0 | **1**(0) | 0 | **20** | **3** | 16% |
| 156 | Pyrococcus furiosus | Archaea | AE009950 | 2125 | 0 | 0 | **5**(0) | 0 | 0 | 0 | 0 | **2**(0) | 0 | **7** | **0** | 0% |
| 157 | Pyrococcus horikoshii | Archaea | BA000001 | 1955 | **1**(0) | **5**(3) | **4**(0) | 0 | 0 | 0 | 0 | **2**(0) | 0 | **12** | **3** | 30% |
| 158 | Pyrococcus abyssi | Archaea | AL096836 | 1896 | **1**(0) | **5**(3) | **4**(0) | 0 | 0 | 0 | 0 | **2**(0) | 0 | **12** | **3** | 30% |
| 159 | Methanothermobacter thermautotrophicus | Archaea | AE000666 | 1873 | **16**(8) | 0 | **3**(1) | 0 | 0 | 0 | 0 | **1**(0) | 0 | **20** | **9** | 47% |
| 160 | Aeropyrum pernix | Archaea | BA000002 | 1841 | 0 | 0 | **4**(1) | **0\*** | 0 | 0 | 0 | **1**(0) | 0 | **5** | **1** | 25% |
| 161 | Methanocaldococcus jannaschii | Archaea | L77117 | 1786 | 0 | 0 | **3**(0) | 0 | 0 | 0 | 0 | **1**(0) | 0 | **4** | **0** | 0% |
| 162 | Methanococcus maripaludis | Archaea | BX950229 | 1722 | **3**(2) | **4**(4) | **4**(0) | 0 | 0 | 0 | 0 | **1**(0) | 0 | **12** | **6** | 55% |
| 163 | Methanopyrus kandleri | Archaea | AE009439 | 1687 | 0 | 0 | **5**(0) | **0\*** | 0 | 0 | 0 | **1**(0) | 0 | **6** | **0** | 0% |
| 164 | Picrophilus torridus | Archaea | AE017261 | 1535 | 0 | 0 | **5**(2) | 0 | 0 | 0 | 0 | 0 | 0 | **5** | **2** | 40% |
| 165 | Thermoplasma volcanium | Archaea | BA000011 | 1499 | 0 | 0 | **4**(2) | 0 | 0 | 0 | 0 | 0 | 0 | **4** | **2** | 50% |
| 166 | Thermoplasma acidophilum | Archaea | AL139299 | 1481 | 0 | 0 | **4**(2) | 0 | 0 | 0 | 0 | 0 | 0 | **4** | **2** | 50% |
| 167 | Nanoarchaeum equitans | Archaea | AE017199 | 535 | 0 | 0 | **3**(0) | 0 | 0 | 0 | 0 | 0 | 0 | **3** | **0** | 0% |

a Within each group, the organisms are sorted by the total number of encoded proteins. The numbers in bold indicate the total number of proteins of each kind, the numbers in parentheses -
the ones with at least one predicted transmembrane segment.
The numbers in the table are linked to protein lists, which are further linked to **RefSeq** and **UniProt** entries, **BLink** outputs,
and **CDD** and **Pfam** domain architectures. Numbers with asterisks indicate the presence of proteins with highly divergent
or truncated domains, not included in the total count but listed at the end of the each protein list. The numbers in the 'Total' column may be smaller than the sum of all types of proteins, as GGDEF and EAL domains are often fused on a single polypeptide chain.
