# Supplementary material for: A census of membrane-bound and intracellular signal transduction proteins in bacteria: Bacterial IQ, extroverts and introverts
Source: BMC Microbiol. 2005 Jun 14;5:35. doi: 10.1186/1471-2180-5-35 (PMC1183210; doi:10.1186/1471-2180-5-35)
Supplement: Additional File 2 — Table 1 in HTML format [file 1471-2180-5-35-S2.html]

Signal census Table 2

**Table 2.� Bacteria with the highest adaptability index ("highest IQ")**

|  |  |  |  |  |
| --- | --- | --- | --- | --- |
| **Organism** | **Phylum** | **Signal  transducers** | **Genome  size, kb** | **IQ** |
| *Wolinella succinogenes* | Epsilon | 99 | 2,110 | 230 |
| *Geobacter sulfurreducens* | Delta | 165 | 3,814 | 166 |
| *Idiomarina loihiensis* | Gamma | 80 | 2,839 | 153 |
| *Desulfovibrio vulgaris* | Delta | 135 | 3,773 | 151 |
| *Vibrio cholerae* | Gamma | 152 | 4,033 | 150 |
| *Thermotoga maritima* | Other | 34 | 1,861 | 145 |
| *Borrelia garinii* | Spiro | 13 | 987 | 143 |
| *Vibrio vulnificus* | Gamma | 200 | 5,127 | 136 |
| *Chromobacterium violaceum* | Beta | 160 | 4,751 | 131 |
| *Thermosynechococcus elongatus* | Cyano | 51 | 2,594 | 131 |
