# Supplementary material for: A census of membrane-bound and intracellular signal transduction proteins in bacteria: Bacterial IQ, extroverts and introverts
Source: BMC Microbiol. 2005 Jun 14;5:35. doi: 10.1186/1471-2180-5-35 (PMC1183210; doi:10.1186/1471-2180-5-35)
Supplement: Additional File 3 — Table 1 in HTML format [file 1471-2180-5-35-S3.html]

Signal census Table 3

**Table 3. Bacteria and archaea with the highest proportion of encoded
signaling proteins of each type**

|  |  |  |
| --- | --- | --- |
| **Organism** | **Phylum** | **No. proteins (%total)** |
| **Histidine kinases** |  |  |
| *Nostoc sp. PCC 7120* | Cyano | 134 (2.2%) |
| *Geobacter sulfurreducens* | Delta | 92 (2.7%) |
| *Bacteroides thetaiotaomicron* | Other | 85 (1.8%) |
| *Rhodopseudomonas palustris* | Alpha | 66 (1.4%) |
| *Desulfovibrio vulgaris* | Delta | 64 (1.8%) |
| *Wolinella succinogenes* | Epsilon | 39 (1.9%) |
| *Haloarcula marismortui* | Archaea | 59 (1.4%) |
| **MCPs** |  |  |
| *Vibrio vulnificus* | Gamma | 52 (1.2%) |
| *Pseudomonas syringae* | Gamma | 48 (0.9%) |
| *Vibrio cholerae* | Gamma | 45 (1.2%) |
| *Chromobacterium violaceum* | Beta | 42 (1.0%) |
| *Clostridium acetobutylicum* | Firmicutes | 38 (1.0%) |
| *Wolinella succinogenes* | Epsilon | 31 (1.5%) |
| *Halobacterium salinarium* | Archaea | 17 (0.6%) |
| **Ser/Thr protein kinases** |  |  |
| *Rhodopirellula baltica* | Other | 60 (0.8%) |
| *Nostoc sp. PCC 7120* | Cyano | 52 (0.9%) |
| *Streptomyces coelicolor* | Actino | 37 (0.4%) |
| *Streptomyces avermitilis* | Actino | 35 (0.4%) |
| *Gloeobacter violaceus* | Cyano | 20 (0.4%) |
| *Thermosynechococcus elongatus* | Cyano | 17 (0.7%) |
| *Sulfolobus tokodaii* | Archaea | 12 (0.4%) |
| **Diguanylate cyclases** |  |  |
| *Vibrio vulnificus* | Gamma | 66 (1.5%) |
| *Shewanella oneidensis* | Gamma | 52 (1.2%) |
| *Vibrio parahaemolyticus* | Gamma | 44 (0.9%) |
| *Chromobacterium violaceum* | Beta | 43 (1.0%) |
| *Vibrio cholerae* | Gamma | 41 (1.1%) |
| *Idiomarina loihiensis* | Gamma | 33 (1.3%) |
| **Adenylate cyclases** |  |  |
| *Bradyrhizobium japonicum* | Alpha | 37 (0.4%) |
| *Sinorhizobium meliloti* | Alpha | 28 (0.5%) |
| *Leptospira interrogans* | Spiro | 18 (0.4%) |
| *Mycobacterium bovis* | Actino | 16 (0.4%) |
| *Mycobacterium tuberculosis* | Actino | 16 (0.4%) |
| *Treponema denticola* | Spiro | 9 (0.3%) |
| **HD-GYP domains** |  |  |
| *Desulfovibrio vulgaris* | Delta | 14 (0.4%) |
| *Vibrio vulnificus* | Gamma | 13 (0.3%) |
| *Chromobacterium violaceum* | Beta | 11 (0.2%) |
| *Thermotoga maritima* | Other | 10 (0.5%) |
| *Desulfotalea psychrophila* | Delta | 10 (0.3%) |
| *Geobacter sulfurreducens* | Delta | 10 (0.3%) |
